# Heat shock protein 90 facilitates SARS-CoV-2 structural protein-mediated virion assembly and promotes virus-induced pyroptosis

**DOI:** 10.1016/j.jbc.2023.104668

**Published:** 2023-04-01

**Authors:** Zhuangzhuang Zhao, Ling-Dong Xu, Fei Zhang, Qi-Zhang Liang, Yajuan Jiao, Fang-Shu Shi, Biao He, Pinglong Xu, Yao-Wei Huang

**Affiliations:** 1 Guangdong Laboratory for Lingnan Modern Agriculture, College of Veterinary Medicine, South China Agricultural University, Guangzhou, China; 2 Department of Veterinary Medicine, Zhejiang University, Hangzhou, China; 3 MOE Laboratory of Biosystems Homeostasis & Protection and Innovation Center for Cell Signaling Network, Life Sciences Institute, Zhejiang University, Hangzhou, China; 4 Changchun Veterinary Research Institute, Chinese Academy of Agricultural Sciences, Changchun, Jilin Province, China

**Keywords:** SARS-CoV-2, Hsp90, nucleoprotein, degradation, E3 ligase, gasdermin D, pyroptosis

## Abstract

Inhibition of heat shock protein 90 (Hsp90), a prominent molecular chaperone, effectively limits severe acute respiratory syndrome coronavirus 2 (SARS-CoV-2) infection but little is known about any interaction between Hsp90 and SARS-CoV-2 proteins. Here, we systematically analyzed the effects of the chaperone isoforms Hsp90α and Hsp90β on individual SARS-CoV-2 viral proteins. Five SARS-CoV-2 proteins, namely nucleocapsid (N), membrane (M), and accessory proteins Orf3, Orf7a, and Orf7b were found to be novel clients of Hsp90β in particular. Pharmacological inhibition of Hsp90 with 17-DMAG results in N protein proteasome-dependent degradation. Hsp90 depletion-induced N protein degradation is independent of CHIP, a ubiquitin E3 ligase previously identified for Hsp90 client proteins, but alleviated by FBXO10, an E3 ligase identified by subsequent siRNA screening. We also provide evidence that Hsp90 depletion may suppress SARS-CoV-2 assembly partially through induced M or N degradation. Additionally, we found that GSDMD-mediated pyroptotic cell death triggered by SARS-CoV-2 was mitigated by inhibition of Hsp90. These findings collectively highlight a beneficial role for targeting of Hsp90 during SARS-CoV-2 infection, directly inhibiting virion production and reducing inflammatory injury by preventing the pyroptosis that contributes to severe SARS-CoV-2 disease.

Coronavirus disease 2019 (COVID-19) is caused by severe acute respiratory syndrome coronavirus 2 (SARS-CoV-2). COVID-19 is a highly infectious, severe respiratory disease characterized by fever, cough, sputum production, fatigue, and myalgia (1, 2). More than 542 million cases of COVID-19 have been reported to date, resulting in more than 6.5 million deaths. SARS-CoV-2 infection can trigger the release of various proinflammatory cytokines including tumour necrosis factor-a (TNF-α), interleukin (IL)-1β, or IL-6 that are associated with damage to vital organs during COVID-19 (1, 2, 3).

SARS-CoV-2 (family Coronaviridae, order *Nidovirales*) has a positive-sense, single-stranded RNA genome of about 30 kb with high sequence similarity to SARS-CoV (4). The viral genome encodes nonstructural proteins (NSP1–16), four major structural proteins (spike [S], envelope [E], membrane [M], and nucleocapsid [N]) and accessory proteins (Orf3, Orf6, Orf7a, Orf7b, Orf8, and Orf9b) (5, 6). These proteins make up components of the viral machinery during different steps of the viral life cycle.

Viruses are dependent on the host cell machinery for protein homeostasis or proteostasis. Heat shock protein 90 (Hsp90), one of the most abundantly expressed molecular chaperones in eukaryotic cells, participates in the stabilization and activation of >200 proteins, termed Hsp90 client proteins (7). Hsp90 has two cytosolic isoforms in mammalian cells: the stress-inducible Hsp90α and the constitutively expressed Hsp90β (8). Hsp90 is known to chaperone diverse viral proteins such as polymerase, capsid, and attachment proteins, thereby regulating the viral life cycle. Studies have shown that Hsp90 inhibitors can significantly inhibit SARS-CoV-2 infection in cell lines (9, 10, 11, 12). However, it is still unknown which viral proteins bind to Hsp90 and which phase of the SARS-CoV-2 life cycle they affect.

Recent studies have shown that in addition to decreasing SARS-CoV-2 infection, Hsp90 inhibitors also downregulate expression of virus-induced inflammatory genes, such as IL-6 and nuclear factor κB (10, 12). However, the mechanism by which Hsp90 regulates SARS-CoV-2-induced inflammation is not fully understood. Pyroptosis is a recently discovered inflammatory form of programmed cell death, in which the sensor NLRP3 forms a functional inflammasome complex containing caspase-1 (13). Caspase-1 mediates the processing of IL-1β and IL-18 into their active forms and cleavage of the pore-forming gasdermin D (GSDMD) induces pyroptotic cell death and the release of proinflammatory cytokines (14, 15). The Hsp90 inhibitor geldanamycin is a recently discovered drug that was able to rescue a human monocyte cell line (THP-1) from lipopolysaccharide (LPS)-induced pyroptosis, which may be achieved through the misfolding and degradation of NLRP3 (16, 17). Emerging evidence suggests that SARS-CoV-2 can trigger pyroptosis (18, 19, 20), which is associated with the severe symptoms of COVID-19 (18, 20, 21, 22, 23, 24). Thus, we hypothesized that Hsp90 activity is linked to virus-induced cellular pyroptosis, resulting in a reduction of SARS-CoV-2-mediated expression of proinflammatory cytokines.

Here we address the role of Hsp90 chaperones in the SARS-CoV-2 life cycle. We found five SARS-CoV-2 proteins (N, M, Orf3, Orf7a, and Orf7b) to be Hsp90 client proteins and that Hsp90 facilitates virion assembly. We also showed that Hsp90 inhibitors suppressed SARS-CoV-2-induced cell pyroptosis. The multifaceted role of Hsp90 in SARS-CoV-2 infection may provide a basis for broad-spectrum, resistance-free antivirals for emerging infectious diseases.

## Results

### Hsp90 interacts with SARS-CoV-2 N, M, Orf3, Orf7a, and Orf7b proteins

**Figure 1:**
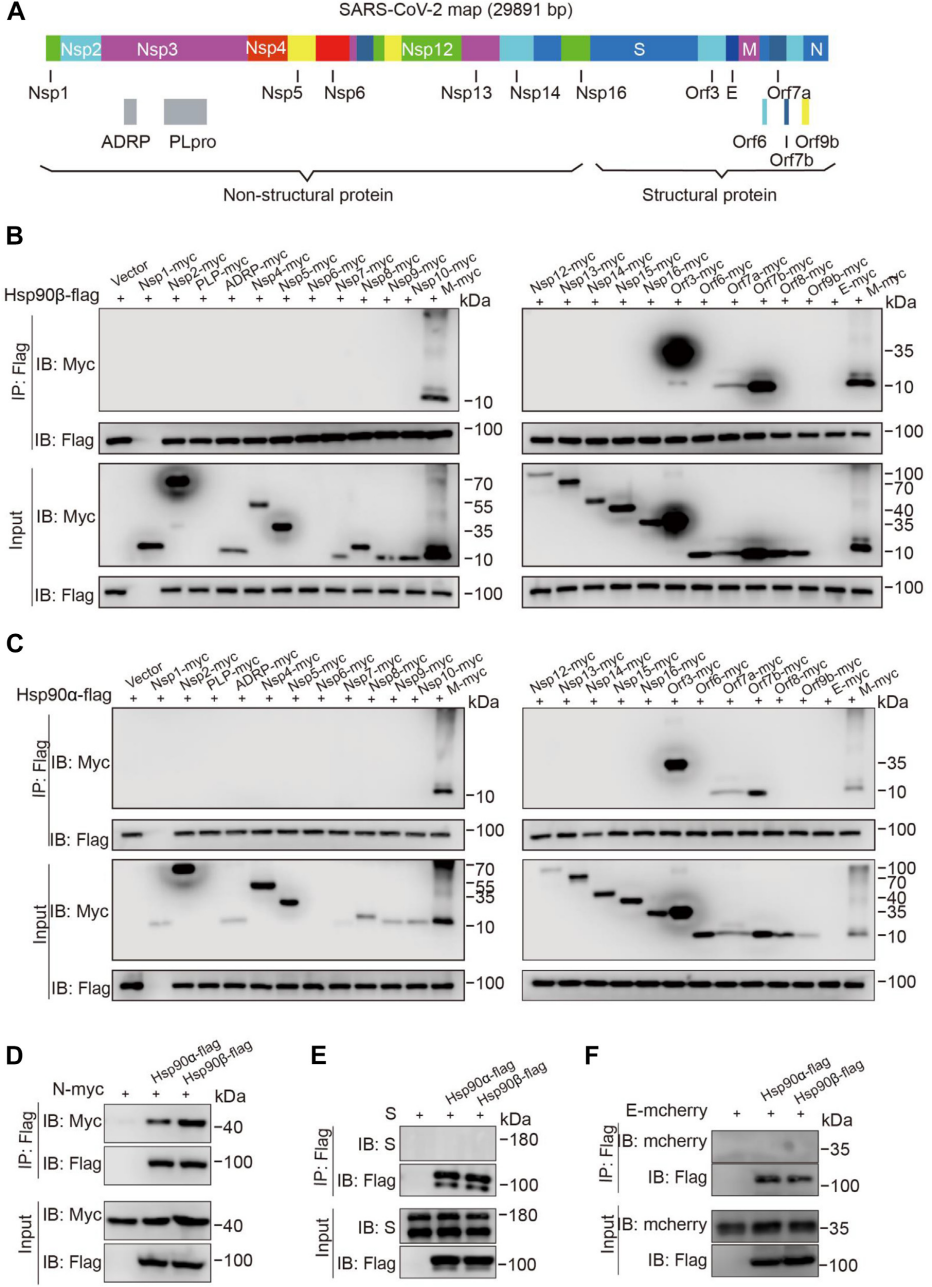
**Screening of Hsp90α/β-interacting proteins among the 26 SARS-CoV-2 viral proteins.***A*, diagram of the SARS-CoV-2 genome and the names of all viral genes. Among the 16 nonstructural proteins (NSPs), NSP3 was dissected into its papain-like protease (PLpro) and ADP-ribose domains (ADRP) domains. The structural proteins are spike (S), membrane (M), envelope (E), and nucleoprotein (N). The accessory proteins are Orf3, Orf6, Orf7a, Orf7b, Orf8, and Orf9b. *B*–*F*, screening of Hsp90α/β interactors by coimmunoprecipitation (CoIP) assay. HEK293T cells were transfected with Hsp90β-flag (*B* and *D*) or Hsp90α-flag (*C* and *D*) vectors and myc-tagged SARS-CoV-2 proteins; total cellular proteins were extracted. CoIP assay was performed using an anti-flag (F3165) antibody and the CoIP protein complexes were screened by Western blot analysis using an anti-myc (71D10) antibody. HEK293T cells were transfected with pRK5-S (*E*) or E-mCherry (*F*) vectors and flag-tagged Hsp90α/β vectors; total cellular proteins were extracted. CoIP assay was performed using an anti-flag (F3165) antibody and the CoIP protein complexes were screened by Western blot analysis using an anti-S or anti-mCherry antibody. Proteins from lysates without pull-down confirmed the presence of the SARS-CoV-2 proteins as appropriate. Hsp90, heat shock protein 90; SARS-CoV-2, severe acute respiratory syndrome coronavirus 2.

Coimmunoprecipitation (CoIP) assays were performed to investigate the interaction between Hsp90α/β and each of 26 of the 30 currently known SARS-CoV-2 proteins with myc-tag (NSP1, NSP2, NSP3 [papain-like protease (PLpro), and ADP-ribose], NSP4, NSP5, NSP6, NSP7, NSP8, NSP9, NSP10, NSP12, NSP13, NSP14, NSP15, NSP16, S, Orf3, E, M, Orf6, Orf7a, Orf7b, Orf8, Orf9b, and N) (Fig. 1*A*). Two proteins, PLpro and NSP6 showed no signal when evaluated in western analysis. To evaluate the interaction of the remaining 24 proteins with Hsp90, HEK293T cells were cotransfected with a single SARS-CoV-2 protein expression vector and Hsp90α-flag or Hsp90β-flag vectors to evaluate their interaction. CoIP bands for Hsp90α and Hsp90β were detected for SARS-CoV-2 M, Orf3, Orf7a, Orf7b (Fig. 1, *B* and *C*), and N protein (Fig. 1*D*). S and E proteins were not seen to interact with either of the Hsp90 isoforms (Fig. 1, *E* and *F*).

**Figure 2:**
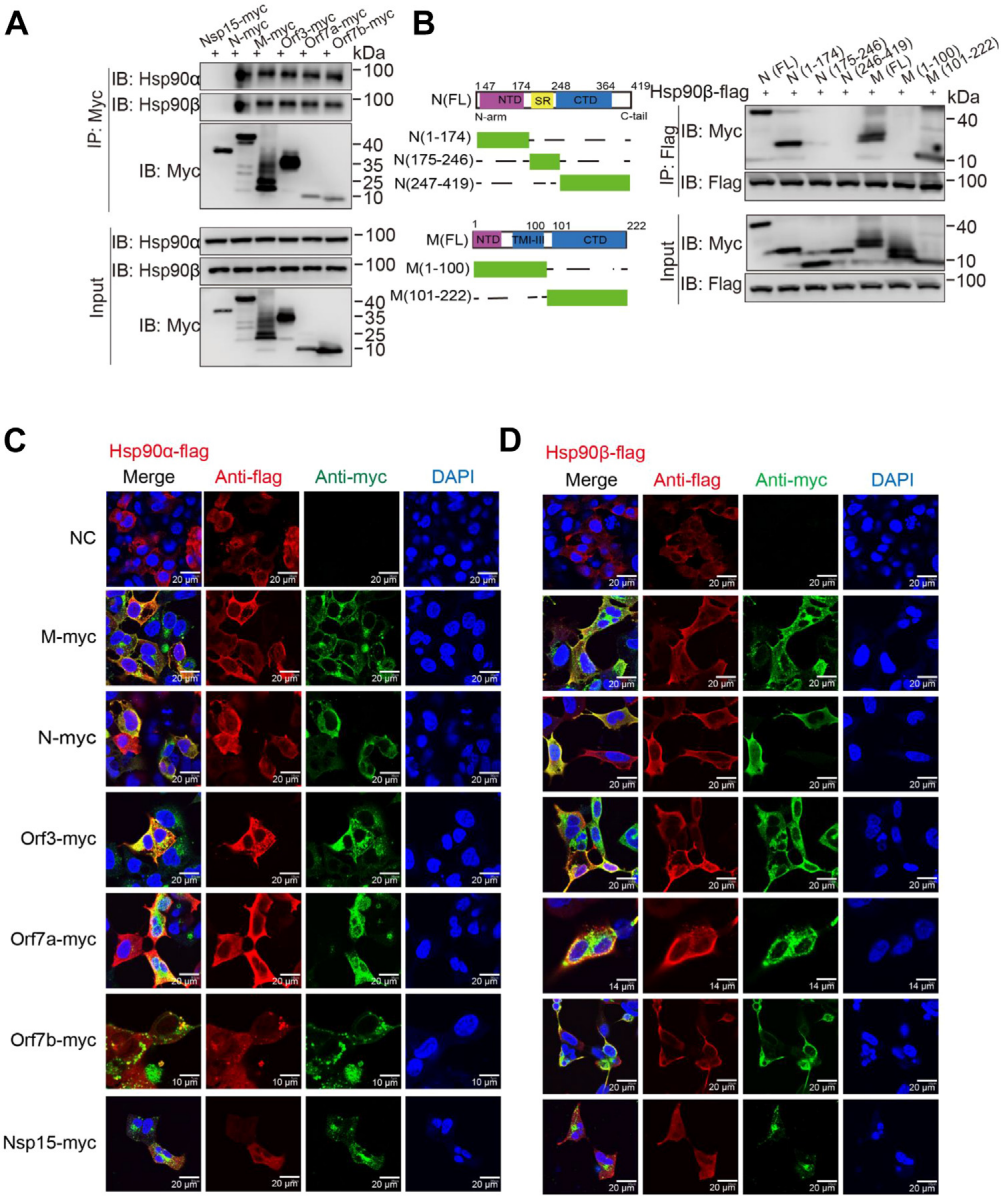
**Colocalization of Hsp90α/β and SARS-CoV-2 Orf7a, Orf7b, Orf3, M, and N proteins.***A*, Western blot analysis of the interaction between SARS-CoV-2 Orf7a, Orf7a, Orf3, M, or N proteins with endogenous Hsp90α/β proteins by reverse coimmunoprecipitation (CoIP) assay. *B*, the functional domains of SARS-CoV-2 M and N proteins; N-terminal domain (NTD), transmembrane region (TM), and C-terminal domain (CTD). HEK293T cells were cotransfected with Hsp90β-flag and myc-tagged WT or truncated M and N expression vectors. CoIP assay was performed using an anti-flag antibody. Colocalization of Hsp90α (*C*) or Hsp90β (*D*) with SARS-CoV-2 Orf7a, Orf7b, Orf3, M, N, and NSP15 proteins. HEK293T cells were grown on coverslips and then transfected with Hsp90β-flag or Hsp90α-flag vectors and myc-tagged SARS-CoV-2 protein vectors. At 24 h post-transfection, cells were fixed, permeabilized, and incubated with primary antibodies directed against flag (Hsp90α/β) and myc (SARS-CoV-2 Orf7a, Orf7b, Orf3, M, and N proteins) tags, followed by incubation with appropriate secondary antibodies. Cell nuclei were subsequently stained with DAPI. Images were collected on a confocal laser scanning microscope. Hsp90, heat shock protein 90; NSP, nonstructural protein; SARS-CoV-2, severe acute respiratory syndrome coronavirus 2.

Reverse CoIP assays were performed using an anti-myc antibody, resulting in visible bands for SARS-CoV-2 N, M, Orf3, Orf7a, and Orf7b associated with both Hsp90α and Hsp90β (Fig. 2*A*). We used CoIP assays to further investigate which functional domain of the SARS-CoV-2 M and N proteins interacts with Hsp90β. We constructed five truncated mutants of viral M and N proteins (M1-100, M101-222, N1-174, N175-247, and N247-419) (Fig. 2*B*) (25, 26). HEK293T cells were cotransfected with Hsp90β-flag and myc-tagged WT or truncated M and N expression vectors. CoIP banding for Hsp90β showed interaction with N protein N terminus (N1-174) and the M protein C terminus (M101-222) (Fig. 2*B*). These results verified the interaction of myc-tagged SARS-CoV-2 proteins with the two Hsp90 isoforms.

### Hsp90α/β colocalize with viral N, M, Orf3, Orf7a, and Orf7b proteins within cells

HEK293T cells were cotransfected with myc-tagged SARS-CoV-2 N, M, Orf3, Orf7a, or Orf7b vectors and flag-tagged Hsp90α or Hsp90β. Colocalization between Hsp90α/β and SARS-CoV-2 N, M, Orf3, Orf7a, or Orf7b proteins was examined by immunofluorescence at 2 days post-transfection. Confocal microscopy revealed a considerable level of colocalization between viral N, M, Orf3, Orf7a, and Orf7b proteins and Hsp90α/β proteins (Fig. 2, *C* and *D*), whereas little colocalization was observed with NSP15 protein (which previously showed no interaction with Hsp90 [Fig. 1, *B* and *C*]). This observation of the subcellular localization of SARS-CoV-2 N, M, Orf3, Orf7a, Orf7b, and NSP15 was consistent with published article (27).

### Hsp90β, but not Hsp90α, regulates SARS-CoV-2 N, M, Orf3, Orf7a, and Orf7b protein levels

**Figure 3:**
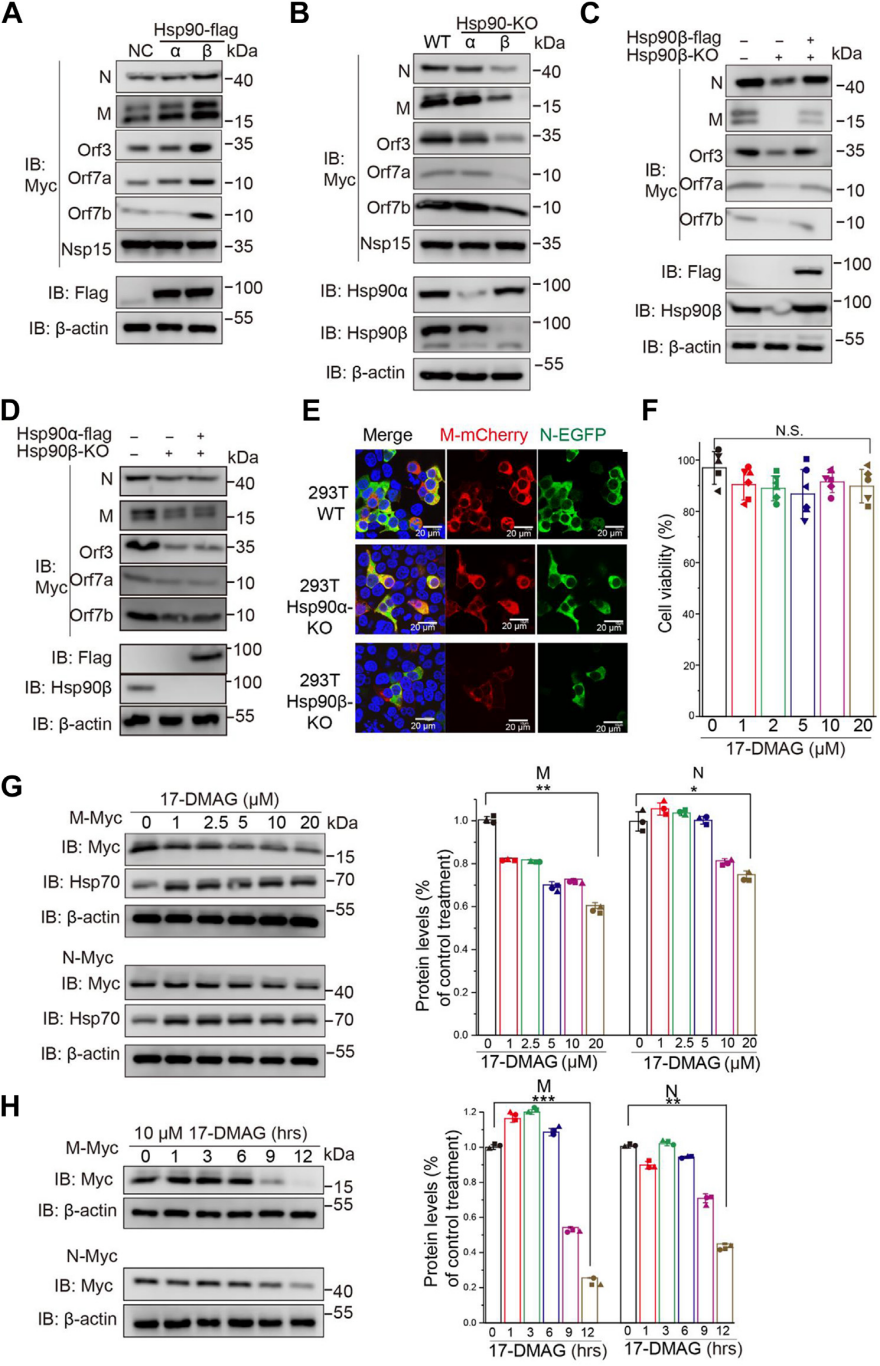
**Inhibition of Hsp90 activity posttranslationally decreases the amount of SARS-CoV2 proteins.** Effect of overexpression (*A*) or knockout (*B*) of two Hsp90 isoforms on SARS-CoV-2 Orf7a, Orf7b, Orf3, M, and N protein expression. *C* and *D*, effect of overexpression of Hsp90β (*C*) or Hsp90α (*D*) on SARS-CoV-2 Orf7a, Orf7b, Orf3, M, and N protein expression in 90β-KO HEK293T cells. *E*, WT, 90α-KO, or 90β-KO HEK293T cells were transfected with M-mCherry or N-GFP vectors for 24 h and observed by fluorescence microscopy. *F*, cytotoxicity was measured in HEK293T cells after 12 h treatment with 17-DMAG. Cell viability was determined and calculated as a percentage of the viable cells after treatment with DMSO. *G*, HEK293T cells were transfected with pRK5-N or pRK5-M, and at 12 h post-transfection (hpt), fresh medium was added containing the indicated concentrations of 17-DMAG. At 24 hpt, cell lysates were subjected to immunoblot with the indicated antibodies. β-actin was used to normalize band intensity and the relative expression level of each viral protein was based on the levels of vehicle (0 μM 17-DMAG)-treated cells. *H*, HEK293T cells were transfected with pRK5-N or pRK5-M and fresh medium containing 10 μM 17-DMAG was added at the indicated times. At 24 hpt, cell lysates were subjected to immunoblot with the indicated antibodies. β-actin was used to normalize band intensity and the relative expression level of each viral protein was based on the levels of DMSO-treated cells; ∗*p* ≤ 0.05; ∗∗*p* ≤ 0.01; n.s.: Not significant. Hsp90, heat shock protein 90; SARS-CoV-2, severe acute respiratory syndrome coronavirus 2.

To determine the relationship between Hsp90α/β and select SARS-CoV-2 proteins, we overexpressed or knocked out Hsp90α/β in HEK293T cells and transfected with SARS-CoV-2 N, M, Orf3, Orf7a, and Orf7b expression vectors. For this purpose, CRISPR-Cas9 was used to create Hsp90α^−/−^ (90α-KO) and Hsp90β^−/−^ (90β-KO) mutant HEK293T cell lines. N, M, Orf3, Orf7a, and Orf7b levels were significantly increased or decreased by overexpression or KO of Hsp90β, respectively, whereas Hsp90α KO had no effect (Fig. 3, *A* and *B*).

To confirm the effect of Hsp90β on viral protein expression, rescue assays were performed in 90β-KO cells by transfection with Hsp90β-flag. SARS-CoV-2 N, M, Orf3, Orf7a, and Orf7b protein expression was restored to normal levels in 90β-KO cells after coexpression of Hsp90β-flag (Fig. 3*C*), and SARS-CoV-2 N, M, Orf3, Orf7a, and Orf7b protein expression was not changed in 90β-KO cells after coexpression of Hsp90α-flag (Fig. 3*D*).

Since the SARS-CoV-2 structural proteins N, M, Orf3, Orf7a, and Orf7b interact with both Hsp90 isoforms, we hypothesized that Hsp90 affects the viral assembly process. Thus, we focused all remaining experiments on the M and N proteins, which are the minimal requirement for SARS-CoV-2 virus-like particle (VLP) formation (28, 29, 30). We also performed binding assays to test whether Hsp90 participates in the interaction between viral M and N proteins in the following study, as M–N interaction is also essential for SARS-CoV-2 virion assembly (31).

### Hsp90 inhibition induces proteasomal degradation of viral N protein

**Figure 4:**
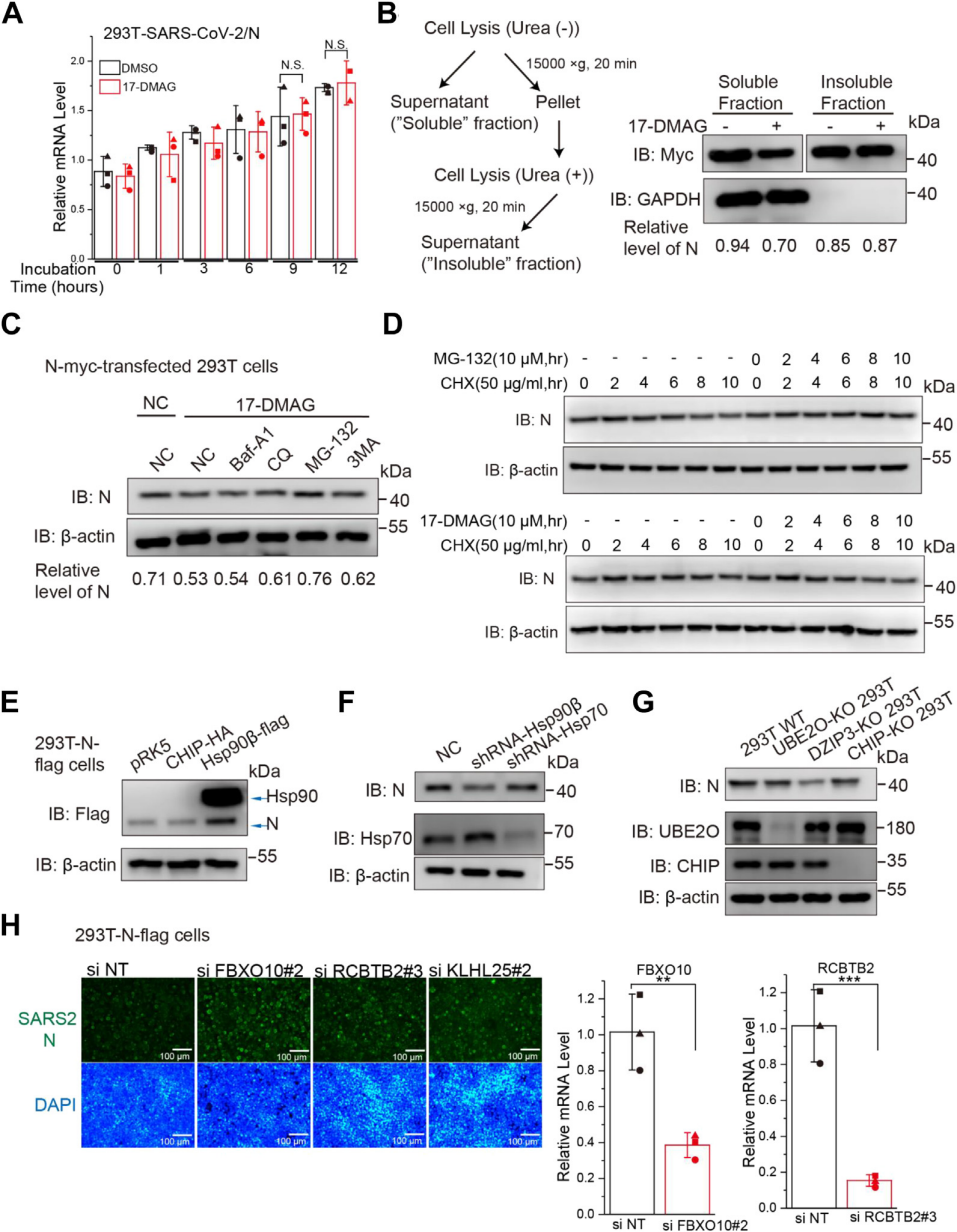
**Hsp90 inhibition mediates proteasomal degradation of viral N protein.***A*, effect of 17-DMAG treatment on viral N mRNA levels, as determined by qRT-PCR; ∗*p* ≤ 0.05; ∗∗*p* ≤ 0.01; n.s.: not significant. *B*, at 24 h post-transfection, soluble and insoluble fractions of the cells were prepared as indicated and subjected to immunoblot, the relative level of N protein was quantified by immunoblot scanning and normalized with respect to the amount of GAPDH. *C* and *D*, HEK293T cells were transfected with N-myc after blocking protein synthesis with cycloheximide (CHX) and the cells were then treated with the protease inhibitor MG132 or the autophagy inhibitor 3-methyladenine (3MA), chloroquine (CQ), or bafilomycin A1 (Baf A1). The relative level of N protein was quantified by immunoblot scanning and normalized with respect to the amount of β-actin. *E*, HEK293T-N-flag cells were transfected with CHIP-HA vector, with Hsp90β-flag vector used as positive control. *F*, HEK293T cells were treated with shRNA-Hsp90β or shRNA-Hsp70 and transfected with N-myc. *G*, WT or CHIP-KO HEK293T cells were transfected with myc-tagged SARS-CoV-2 proteins. *H*, HEK293T-N-flag cells were seeded in 24-well plates and incubated with siRNA for 84 h, cells were immunostained with anti-N (*green*) and nuclei were stained with DAPI (*blue*). The cells were observed by fluorescence microscopy. Intracellular FBXO10 and RCBTB2 mRNA levels were measured by real-time RT-PCR. The data were first normalized to cellular GAPDH mRNA and then to negative control siRNA–treated samples to obtain fold induction. Data are shown as the mean ± SD from three independent experiments. CHIP, C terminus of Hsp70-interacting protein; Hsp90, heat shock protein 90; SARS-CoV-2, severe acute respiratory syndrome coronavirus 2.

Many Hsp90 client proteins are destabilized when Hsp90 is inhibited (32). Thus, we evaluated the effect of Hsp90 inhibition on SARS-CoV-2 M and N protein levels. 17-dimethylaminoethylamino-17-demethoxygeldanamycin (17-DMAG), a potent Hsp90 inhibitor, deactivates Hsp90 by binding to its N-terminal ATP/ADP-binding pocket, which leads to the destabilization and degradation of Hsp90-associated client proteins (33). At noncytotoxic concentrations (Fig. 3*F*), 17-DMAG decreased the expression of viral M and N proteins in a dose- and time-dependent manner (Fig. 3, *E*–*H*), consistent with the Hsp90β KO data (Fig. 3*B*). 17-DMAG treatment had little effect on viral N mRNA level (Fig. 4*A*). We examined the effect of 17-DMAG on the stability of viral N, to determine whether the lower protein level was due to degradation or aggregation in the insoluble fraction. Protein levels were analyzed in soluble and insoluble cell fractions (Fig. 4*B*), with the amounts of SARS-CoV-2 N in insoluble fractions remaining unchanged after 17-DMAG treatment (Fig. 4*B*). These data suggested that the viral proteins were degraded and not aggregated when Hsp90 activity was inhibited.

The ubiquitin-proteasome system and autophagy are two major intracellular protein degradation pathways in eukaryotic cells (34). In order to identify the system that primarily mediates the observed degradation of viral proteins when Hsp90 is inhibited, protein expression was measured in the presence of specific inhibitors of both degradation pathways. HEK293T cells were transfected with N-myc and then treated with the proteasome inhibitor MG132 or the autophagy inhibitor 3-methyladenine, chloroquine, or bafilomycin A1. Degradation of viral N protein mediated by Hsp90 inhibition was blocked by the proteasome inhibitor MG132 (Fig. 4*C*). When cycloheximide was used to block new protein synthesis, viral N protein degradation was significantly enhanced upon treatment with 17-DMAG, or blocked by the proteasome inhibitor MG132 (Fig. 4*D*). Collectively, these data indicate that Hsp90 inhibition triggers proteasomal degradation of SARS-CoV-2 N protein.

### Hsp90 inhibition-induced N protein degradation is independent of CHIP but alleviated by FBXO10

E3 ligases provide target specificity for the ubiquitinylation process, particularly C terminus of Hsp70-interacting protein (CHIP), which bind to Hsp90 (32). First, we tested whether CHIP mediates degradation of viral N protein. Ectopic overexpression of CHIP had no effect on N protein expression in 293T-N-flag stable cells (Fig. 4*E*) nor did CHIP-KO or shRNA knockdown of Hsp70 affect expression of N protein (Fig. 4, *F* and *G*). To identify if another E3 ligase is involved in Hsp90 KO-induced viral protein degradation, we performed an siRNA-based screen targeting human E3 ubiquitin ligases that can bind to Hsp90 (Table S2). To ensure an siRNA that can efficiently induce gene knockdown, the siRNA pools were composed of three sequence-independent siRNAs per gene target (35). Transfection of Hsp90β-flag was used to rescue 90β-KO-N-flag stable cells to serve as a positive control (Fig. S1). The Western blot results revealed that transfection of several potent siRNAs or Hsp90β-flag upregulated the N protein level in 90β-KO-N-flag stable cells. The potency of siRNAs was further confirmed by using SARS-CoV-2 N protein immunofluorescence assay (Fig. S2). The most potent appeared to be siRNAs targeting FBXO10 (F-Box Protein 10) and RCBTB2 (RCC1 and BTB domain-containing protein 2), which upregulated the N protein level in 90β-KO-N-flag stable cells (Fig. 4*H*). FBXO10 gene knockdown was the most consistently effective in Western blot and immunofluorescence (Figs. S1 and S2). The efficiency of FBXO10 or RCBTB2 gene knockdown was measured by the percentage of target mRNA reduction in siRNA-transfected cells relative to negative control siRNA (Fig. 4*H*).

### Hsp90 dysfunction does not alter interactions of M–N and N–RNA

**Figure 5:**
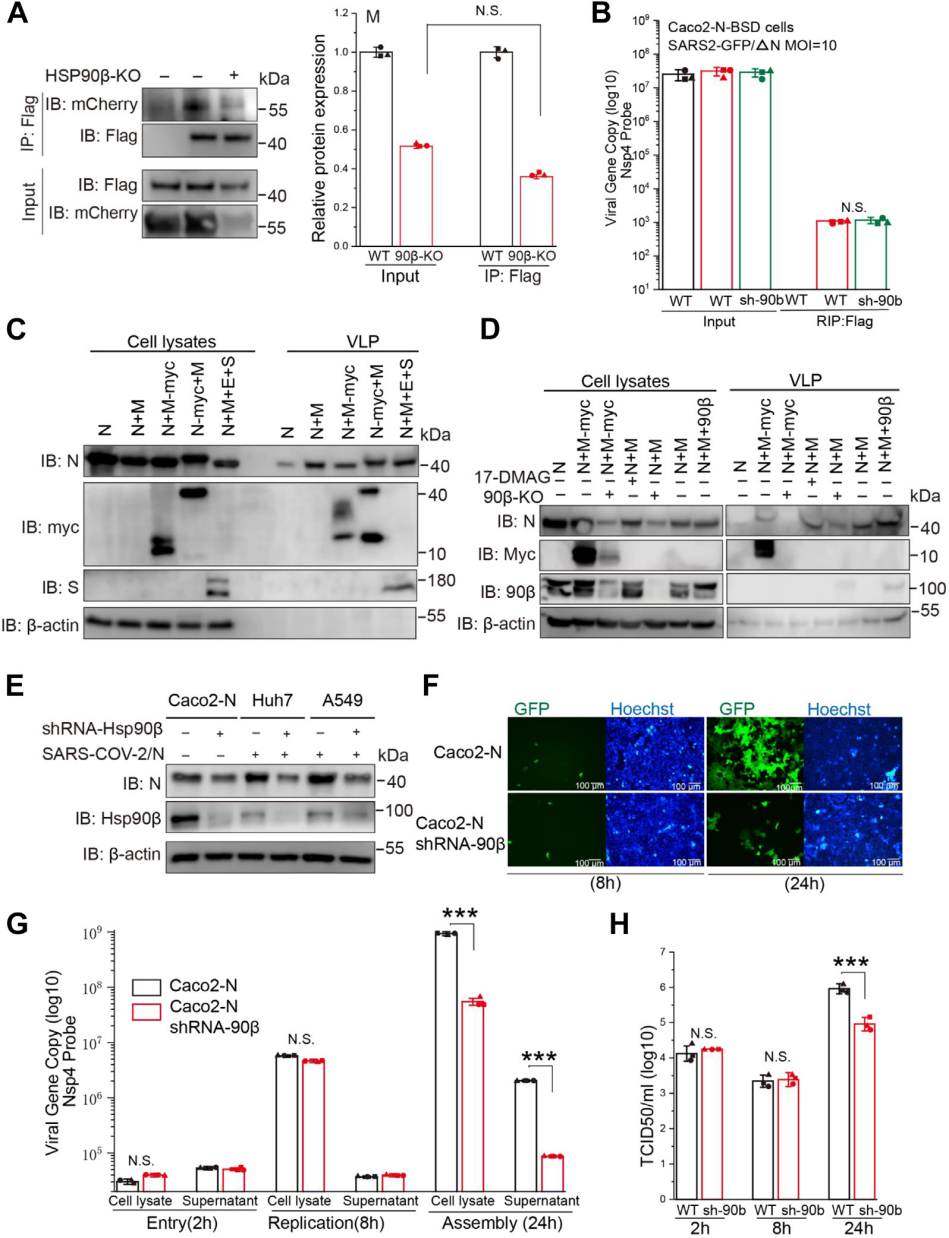
**Disruption of Hsp90 function diminishes efficient virion assembly of SARS-CoV-2.***A*, WT or 90β-KO HEK293T cells were transfected with M and N vectors and total cellular proteins were extracted. CoIP assay was performed using an anti-flag antibody. *B*, Caco-2-N-flag cells or Caco-2-N-flag shRNA-Hsp90β cells were infected with SARS-CoV-2 GFP/ΔN virus at MOI = 1 for 8 h, and total cellular proteins were extracted. RNA immunoprecipitation was performed using an anti-flag antibody. *C*, SARS-CoV-2 VLP assembly and release. HEK293T cells were transfected with M, N, M-tagged myc (M-myc), N-tagged myc (N-myc) expression vectors individually or in various combinations. At 48 h post-transfection, supernatants and cells were collected and prepared for protein analysis as described in the Experimental procedures. *D*, WT and 90β-KO HEK293T cells were transfected with the indicated plasmids for 36 h. Lysates and corresponding purified VLPs were analyzed by Western blot. *E*, Hsp90β knockdown decreases SARS-CoV-2 protein levels in various cell lines (Caco-2-N, Huh7, and A549). *F*–*H*, Caco-2-N cells were infected with SARS-CoV-2 GFP/ΔN virus at MOI = 1. Viral load was detected in cell lysates or supernatant by one-step qRT-PCR at the indicated h postinfection. Viral titration was detected in supernatant by TCID_50_ assay; ∗*p* ≤ 0.05; ∗∗*p* ≤ 0.01; n.s.: Not significant. CoIP, coimmunoprecipitation; Hsp90, heat shock protein 90; SARS-CoV-2, severe acute respiratory syndrome coronavirus 2; TCID_50_, tissue culture ID_50_; VLP, virus-like particle.

Since we observed that Hsp90 dysfunction decreased the subcellular colocalization of M and N (Fig. 3*E*), M–N interaction has been clearly established and is essential for SARS-CoV-2 virion assembly (31). We wanted to further investigate whether Hsp90 participates in the interaction between viral M and N proteins. To address this issue, CoIP assays were performed. WT or 90β-KO HEK293T cells were cotransfected with M-mCherry and N-Flag, and total cellular proteins were extracted. In CoIP bands, M protein interaction with N protein could still be detected after Hsp90β KO (Fig. 5*A*).

As aforementioned, the N-terminal region (NTD) of N interacts with Hsp90β (Fig. 2*B*) and previous studies have revealed that SARS-CoV-2 RNA can binding to either Hsp90β (36) or the N-terminal region (NTD) of N (37). We investigate whether Hsp90 participates in viral RNA and N interaction. RNA immunoprecipitation (RIP) assay indicated that Hsp90β-specific shRNA knockdown had little effect on viral N–RNA interactions in SARS-CoV-2 GFP/ΔN virus infected Caco-2-N cells (Fig. 5*B*).

### Hsp90 facilitates M/N protein-mediated VLP assembly

Considering that expression of viral M and N proteins is the minimal requirement for SARS-CoV-2 VLP formation (28), we began to investigate the effect of Hsp90 on SARS-CoV-2 VLP assembly. We started by validating our system of VLP formation. Following transient expression of N alone, M/N or M/N/E/S from SARS-CoV-2, culture media were collected and subjected to ultracentrifugation to pellet VLPs. When expressed alone, N protein was readily detectable in the cell lysate and hardly at all in medium (supernatant) (Fig. 5*C*). N protein coexpressed with untagged M protein resulted in significant secretion of N in culture supernatants, transient expression of M/N/E/S also resulted in significant secretion of N in culture supernatant (Fig. 5*C*), supporting the requirement of M and N for efficient assembly and release of SARS-CoV-2 VLPs. We used this convenient system to further investigate the mechanism of Hsp90 action on SARS-CoV-2 virion assembly. Employing gene KO, gene overexpression, and Hsp90 inhibition, we found that Hsp90 dysfunction significantly reduced M/N protein-mediated VLP assembly and release (Fig. 5*D*).

### Hsp90 is associated with the later stages of SARS-CoV-2 infection

Next, we used a transcomplementation SARS-CoV-2 cell culture system to examine the involvement of Hsp90 in the SARS-CoV-2 life cycle (38). First, Hsp90β-specific shRNA knockdown was established in Huh-7, Caco-2, and A549 cells, which are susceptible to SARS-CoV-2 infection. As expected, a reduction in N production was observed after Hsp90β knockdown (Fig. 5*E*). The recombinant SARS-CoV2 GFP/ΔN virus, lacking the N gene, can propagate in Caco-2 cells transduced with lentivirus expressing the viral N protein (Caco-2-N) (38). Caco-2-N cells were infected with SARS-CoV-2 GFP/ΔN virus at a multiplicity of infection (MOI) = 1 for 2, 8, and 24 h. Hsp90β-specific shRNA knockdown significantly decreased GFP (Fig. 5*F*), viral RNA (Fig. 5*G*), and viral titer (Fig. 5*H*) at the late phase of infection (24 hpi) but not at the early phase (2 and 8 hpi). Since SARS-CoV-2 virions were assembled greatly at this time point compared to 2 hpi (corresponding to virus entry) and 8 hpi (corresponding to completion of one-round of virus life cycle), these results suggest that Hsp90 depletion is associated with the later stages of SARS-CoV-2 infection and may suppress SARS-CoV-2 assembly in part through its induced M/N degradation.

### Hsp90 inhibition alleviates the pyroptotic cell death triggered by SARS-CoV-2

**Figure 6:**
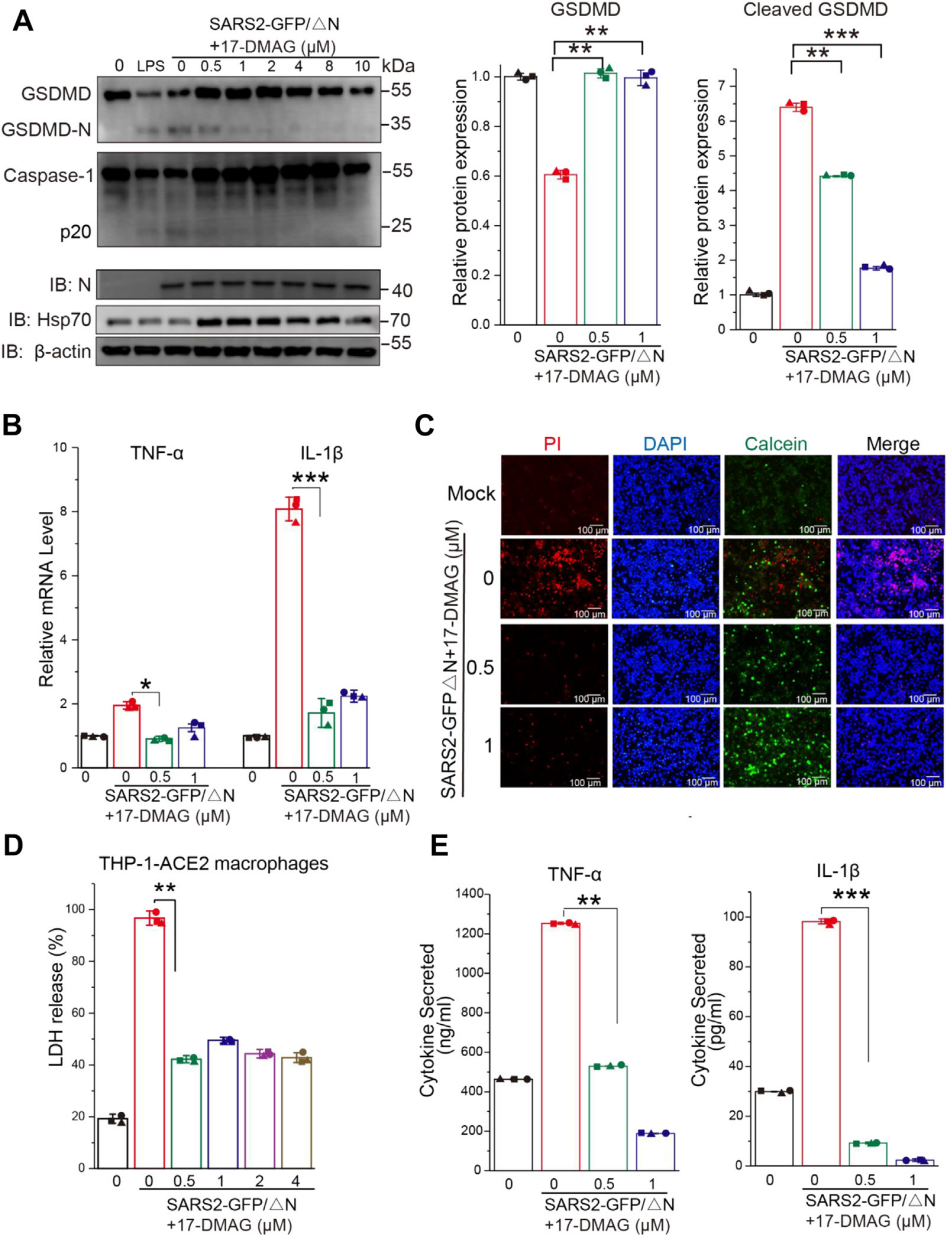
**SARS-CoV-2 infection triggers GSDMD-mediated pyroptosis, which was reduced by inhibition of Hsp90.** THP-1-ACE2 macrophages were infected with SARS-CoV-2 GFP/ΔN virus at an MOI = 10 for 4 h in the presence 17-DMAG. *A*, cells were lysed and levels of caspase-1, GSDMD, cleaved GSDMD, Hsp70, SARS-CoV-2 N, and β-actin were determined by Western blot. The protein level of GSDMD and cleaved GSDMD were quantified by immunoblot scanning and normalized with respect to β-actin. *B*, qRT-PCR for quantification of TNF-α and IL-1β mRNA in THP-1-ACE2 macrophages. *C*, cells were stained with Calcein/PI and DAPI, or tested by LDH assay (*D*). *E*, ELISA was performed to determine IL-1β and TNF-α levels in cell supernatants; ∗*p* ≤ 0.05; ∗∗*p* ≤ 0.01; n.s.: Not significant. ACE2, angiotensin converting enzyme 2; Hsp90, heat shock protein 90; LDH, lactate dehydrogenase; SARS-CoV-2, severe acute respiratory syndrome coronavirus 2.

Previous studies have suggested that SARS-CoV-2 can cleave GSDMD (18, 20, 23), which triggers pyroptosis, but it is unknown whether Hsp90 is linked to virus-induced cellular pyroptosis. Pyroptosis is a caspase-1-dependent event that leads to GSDMD pore formation (39). We examined whether SARS-CoV-2 could activate GSDMD-mediated pyroptosis in THP-1 expressing angiotensin-converting enzyme 2 (ACE2) macrophages. THP-1-ACE2–derived macrophages were infected with SARS-CoV-2 GFP/ΔN virus at an MOI = 10 for 5 h post pretreatment with 17-DMAG for 12 h. Western blot revealed reduced caspase-1 and cleaved GSDMD bands in SARS-CoV-2-infected macrophages, which were mitigated in intensity by treatment with 17-DMAG (a short time of treatment that did not affect viral replication according to N protein levels) (Fig. 6*A*). Moreover, we found that infection with SARS-CoV-2 GFP/ΔN induced inflammatory cytokines such as IL-1β and TNF-α mRNA expression, all of which could be prevented by 17-DMAG (Fig. 6*B*). 17-DMAG suppressed virus-induced cell death as detected by lactate dehydrogenase assay and Calcein/PI cell viability/cytotoxicity assay (Fig. 6, *C* and *D*). These data demonstrate that 17-DMAG ameliorates the caspase-1/GSDMD-mediated pyroptosis in THP-1-ACE2 macrophages induced by SARS-CoV-2.

The pores formed by activated GSDMD connect the material inside and outside of cells, leading to eventual swelling and breakage that releases proinflammatory cytokines (40, 41). We surveyed the spectrum of secreted cytokines in SARS-CoV-2–infected monocytes through an ELISA array. We found supernatant IL-1β and TNF-α levels in virus-infected cells were higher than those in mock-infected cells. 17-DMAG treatment reduced the secretion of IL-1β and TNF-α (Fig. 6*E*). These data show that inhibition of Hsp90 ameliorates virus-induced pyroptosis in THP-1 macrophages.

### Inhibition of Hsp90 alleviates COVID-19 lung injury in a Syrian hamster model

Having shown that the Hsp90 inhibitor 17-DMAG decreases SARS-CoV-2 replication and associated pyroptosis *in vitro*, we wanted to determine whether it would be effective as a COVID-19 treatment *in vivo*. We carried out animal studies in Syrian hamsters to mirror COVID-19, as described previously (42). Here, we performed intratracheal injection of SARS-CoV-2 GFP/ΔN virus in Syrian hamsters to mirror COVID-19.

**Figure 7:**
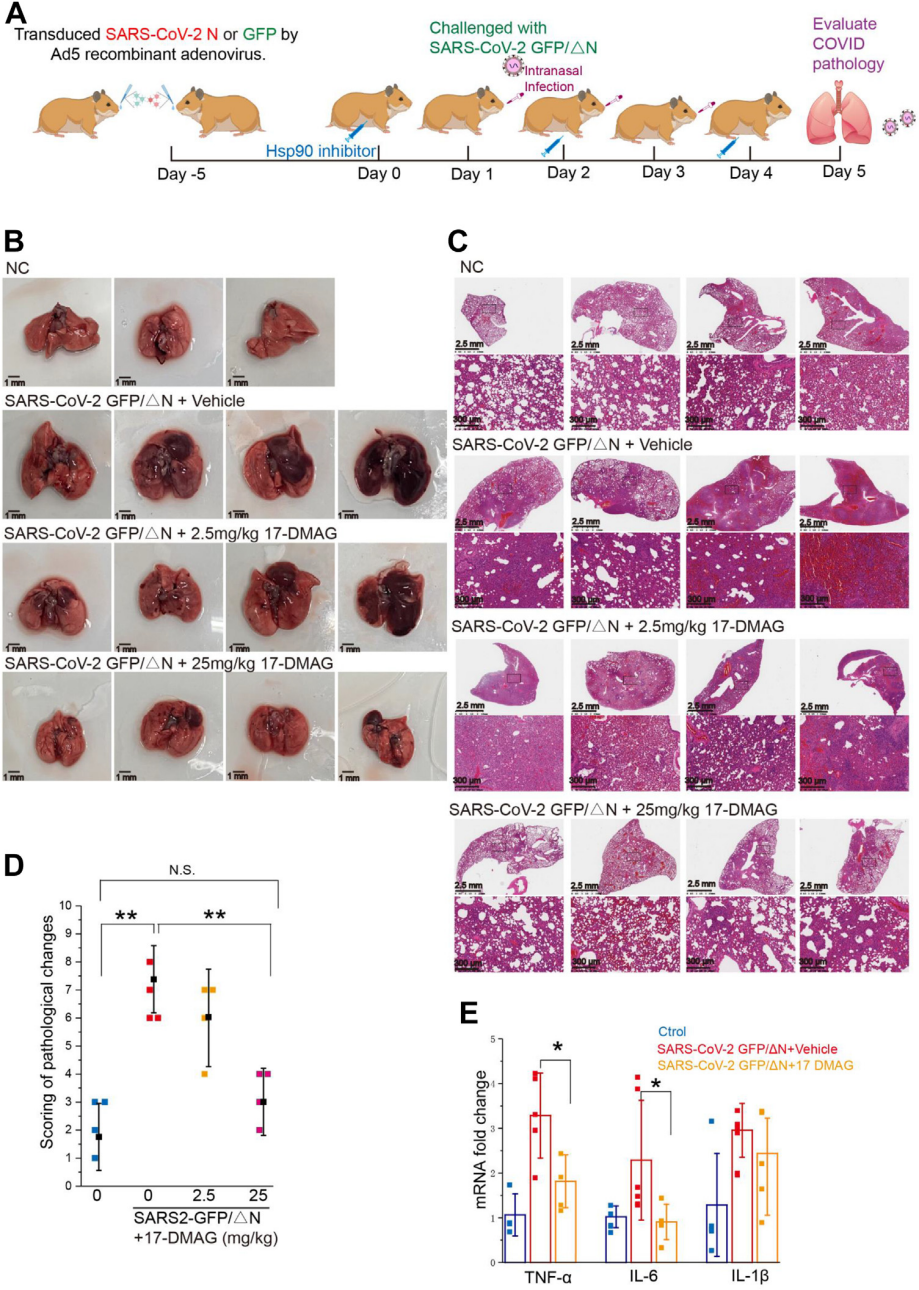
**Hsp90 inhibition alleviates COVID-19 lung injury in a Syrian hamster model.***A*, Ad5-N/GFP-transduced hamsters were intranasally infected with 10^6.5^ pfu/mL of SARS-CoV-2 GFP/ΔN virus. Animals were euthanized at 5 days postinfection (dpi) and lungs were harvested. *B*, photographs of gross pathology in lung specimens isolated from infected hamsters at 5 dpi. *C*, representative H&E staining of lungs from hamsters. *D*, histology scores determined from (*C*). *E*, qRT–PCR was used to quantify transcripts of key inflammatory factors in cytokine storms: TNF-α, IL-6, and IL-1β. Relative expression of target genes was normalized to GAPDH (n = 4–5 hamsters per group); ∗*p* ≤ 0.05; ∗∗*p* ≤ 0.01; n.s.: Not significant. COVID-19, coronavirus disease 2019; Hsp90, heat shock protein 90; SARS-CoV-2, severe acute respiratory syndrome coronavirus 2.

To permit viral propagation in hamsters, viral N protein was provided by exogenous delivery using a replication-deficient adenovirus (Ad5-N). This strategy was first validated *in vitro*, with high viral GFP signal detected in A549-ACE2 and Huh7-ACE2 cells only after transduction with Ad5-N (Fig. S3*A*). After intranasal inoculation of 4-week-old hamsters with 10^8.5^ plaque-forming units (pfu)/ml Ad5-N, we observed N protein expression in the lung (Fig. S3*A*). This convenient model was used to test whether Hsp90 inhibitors are effective as COVID-19 treatment *in vivo*, according to the experimental strategy shown in Figure 7*A*. Quantitative reverse transcription PCR was used to measure SARS-CoV-2 GFP/ΔN replication (NSP4 mRNA) and transcription (M mRNA) (Fig. S3*C*). The viral genomes and M protein transcripts were much lower in the lungs than authentic SARS-CoV-2 infection in hamsters, viral reduction during infection showed no signal when evaluated by the viral titration, indicating recombinant SARS-CoV-2 GFP/ΔN virus did not expand and propagate in our Ad5-N–sensitized hamsters but resulted in only a single-cycle infection in hamsters. However, the infected hamsters did develop labored breathing (difficulty breathing, more rapid breathing rate than usual) (43), lost 8% body weight (Fig. S3*B*), and exhibited a significant increase in lung injury scores (Fig. 7, *B*–*D*). Cytokine expression was upregulated for IL-6, TNF-α, and IL-1β in the small intestine of infected hamsters (Fig. 7*E*).

In 17-DMAG–treated animals, virus-induced gross pathology and histopathology exhibited marked improvement (Fig. 7, *B* and *C*), in particular in the high dose (25 mg/kg) of 17-DMAG, whose lung pathology score was comparable to the baseline score of untreated, noninfected hamsters (*p* > 0.05) (Fig. 7*D*). The high dose (25 mg/kg) of 17-DMAG significantly reduced lung pathology but there were signs of toxicity including ruffled fur and a loss of 20% body weight (Fig. S3*B*). To clearly understand how the SARS-CoV-2 GFP/ΔN virus attacks the lungs of hamsters, we checked various factors released during cytokine storm and noted that TNF-α, IL-6, and IL-1β were upregulated by SARS-CoV-2 GFP/ΔN virus treatment. 17-DMAG alleviated this upregulation of TNF-α, IL-6, and IL-1β (Fig. 7*E*), suggesting its direct role in reduction of inflammation injury in hamsters.

## Discussion

Like all viruses, CoVs subvert the host cellular machinery to support their life cycle. For example, Hsp90α is a component of the receptor complex vital for dengue virus entry (44). Hsp90β has been associated with enterovirus 71 capsid proteins and is needed for viral entry (45). Hsp90 is critical for maintaining activity and stability of the NS2/3 protease, which is essential for RNA replication of hepatitis C virus (46). In CoVs, Hsp90 has been verified to interacts with the N protein of Middle East respiratory syndrome (MERS)-CoV and prevents it from being degraded by proteasomes (9). However, it is unknown which SARS-CoV-2 proteins bind to Hsp90 and which phase of the SARS-CoV-2 life cycle they affect.

Based on our results, we can conclude that SARS-CoV-2 N, M, Orf3, Orf7a, and Orf7b proteins are Hsp90 client proteins and that their expression levels are regulated by the constitutively expressed isoform Hsp90β rather than the inducible Hsp90α (Fig. 3, *A* and *B*). Several studies compared Hsp90α *versus* Hsp90β side-by-side in various viral proteins. MERS-CoV N protein interacted with both Hsp90α and Hsp90β but Hsp90β played a more important role than Hsp90α in the stability of MERS-CoV N protein (9). Herpes simplex virus type 1 virion protein 16 interacted with Hsp90 and Hsp90α played a more important role than Hsp90β in the degradation of virion protein 16 (47). Apart from viral proteins, Hsp90α and Hsp90β also exhibit similar interactions with cochaperones but different behaviors with client proteins in mammalian cells (48, 49). The reason Hsp90α and Hsp90β exhibit different behaviors with client proteins is not clear. Hsp90α has been shown to support tumor progression and substitutions of gly-262 and thr-269 in Hsp90β with lysines convert Hsp90β to an Hsp90α-like protein (50), which may help explain different behaviors in Hsp90 isoforms. The precise reason Hsp90α and Hsp90β exhibit different behaviors with SARS-CoV-2 client proteins needs further investigation. Hsp90 inhibition can induce proteasomal degradation of SARS-CoV-2 N protein. Recent studies have shown that Hsp90β interacts with MERS-CoV N protein (9), although the amino acid homology of MERS-CoV N protein with SARS-CoV-2 N protein is only around 50%. Hsp90 may be a broad-spectrum chaperone for CoV N proteins (51) and indeed we detected interaction of Hsp90β with the N proteins from transmissible gastroenteritis virus, porcine epidemic diarrhea virus, swine acute diarrhea syndrome-CoV, and porcine deltacoronavirus (data not shown).

Furthermore, we found that Hsp90 dysfunction induces proteasome-mediated degradation of SARS-CoV-2 N protein. Our results suggest that Hsp90 dysfunction-induced N protein degradation is independent of CHIP. Previous quantitative analysis of Hsp90 clients revealed that about 30% of human E3 ubiquitin ligases were found to bind to Hsp90 (52). Our siRNA-based screen of human E3 ubiquitin ligases that can bind to Hsp90 discovered that FBXO10 was linked to N-protein degradation in 90β-KO cells.

In addition to changes in protein level, we explored the effect of Hsp90 inhibition/KO on the functions of SARS-CoV-2 M and N proteins. CoV N proteins are typically composed of three distinct but highly conserved components: an N-terminal RNA-binding domain (NTD); a C-terminal domain; and a poorly structured central Ser/Arg-rich linker. We found that Hsp90β interacts with the NTD of N protein and the C-terminal region of M protein. Previous studies have revealed that the NTD of N protein is responsible for SARS-CoV-2 RNA binding to N protein (37), and SARS-CoV-2 RNA can bind to Hsp90α and Hsp90β (36); however, the result from RIP assay in this study showed Hsp90 knockdown did not alter N–RNA interactions. In addition to binding the RNA of the virus particle, SARS-CoV-2 M and N proteins also bind to each other (25, 26, 31). Condensation of the N protein forms with the M protein is crucial because it mediates packaging of the viral RNA–protein (vRNP) complex into virions (26, 31, 53). These multiple interactions enable virus assembly and CoIP assays showed no change in N–M interactions after Hsp90 gene KO, suggesting that Hsp90 exerts its effect on viral assembly exclusively through regulation of M and N protein levels.

Each of the novel Hsp90 SARS-CoV-2 protein clients (N, M, Orf3, Orf7a, and Orf7b) described herein are viral structural proteins. Given that CoV structural proteins S, E, M, and N bud and assemble at the endoplasmic reticulum-Golgi intermediate compartment, we hypothesized that Hsp90 affects the assembly process of the virus. We found that Hsp90 KO or pharmaceutical targeting significantly reduced the efficiency of VLP assembly, while overexpression of Hsp90 increased VLP formation (Fig. 5*D*). In a transcomplement cell culture model of SARS-CoV-2, Hsp90 knockdown in Caco-2-N cells reduced the SARS-CoV-2 GFP/ΔN virus assembly process but did not affect virus entry or replication. Thus, it seems that Hsp90 directly facilitates SARS-CoV-2 infection at the virus assembly stage *via* regulation of M and N protein levels.

Pyroptosis is a recently discovered inflammatory form of programmed cell death and emerging evidence suggests that SARS-CoV-2 triggers pyroptosis in cases of severe disease (18, 19, 20). GSDMD-mediated pore formation results in pyroptosis, releasing various inflammatory cytokines (54). We found that the Hsp90 inhibitor 17-DMAG can suppress macrophage cell death, active caspase-1, and GSDMD cleavage after SARS-CoV-2 GFP/ΔN infection. Among the proteins that Hsp90 chaperoned, N protein has a significant effect on pyroptosis, while M, Orf3, and Orf7a have a relatively weak effect (20, 24) and there are no published reports on the effect of Orf7b on pyroptosis. In our protocol, cells were infected with SARS-CoV-2 for 5 h, in which case the effect of Hsp90 on N protein was reduced to very low levels, according to the N protein expression in these cells (Fig. 6*A*). As for M, Orf3, Orf7a, and Orf7b proteins, as mentioned above, their effect on pyroptosis was relatively weaker than that of N protein. The effect of Hsp90 on SARS-CoV-2-triggered pyroptosis is more likely direct and not an indirect consequence of a general reduction in viral proteins due to Hsp90 inhibition. Inhibition of Hsp90 has been shown previously to downregulate the expression of SARS-CoV-2-induced proinflammatory cytokines (10, 12). This may be explained by our finding that inhibition of Hsp90 mitigated GSDMD-mediated pyroptotic cell death triggered by SARS-CoV-2 infection.

Although studies have shown that Hsp90 inhibitors can significantly inhibit SARS-CoV-2 infection in cell lines (9, 10, 11, 12), *in vivo* studies are lacking. We found that intranasal inoculation of Syrian hamsters with high-dose SARS-CoV-2 GFP/ΔN virus caused severe lung injury with similar characteristics to WT authentic SARS-CoV-2 infection (42, 55). Treatment with the Hsp90 inhibitor 17-DMAG significantly reduced gross and microscopic lesions in the lungs of infected hamsters, though some evidence of toxicity indicates the potential limitations of Hsp90 inhibitors for treatment of COVID-19. This phenomenon also exists in other diseases, such as lipid disorders (56, 57). The search for Hsp90 inhibitors with lower toxicity as antivirals is needed in the future (58).

**Figure 8:**
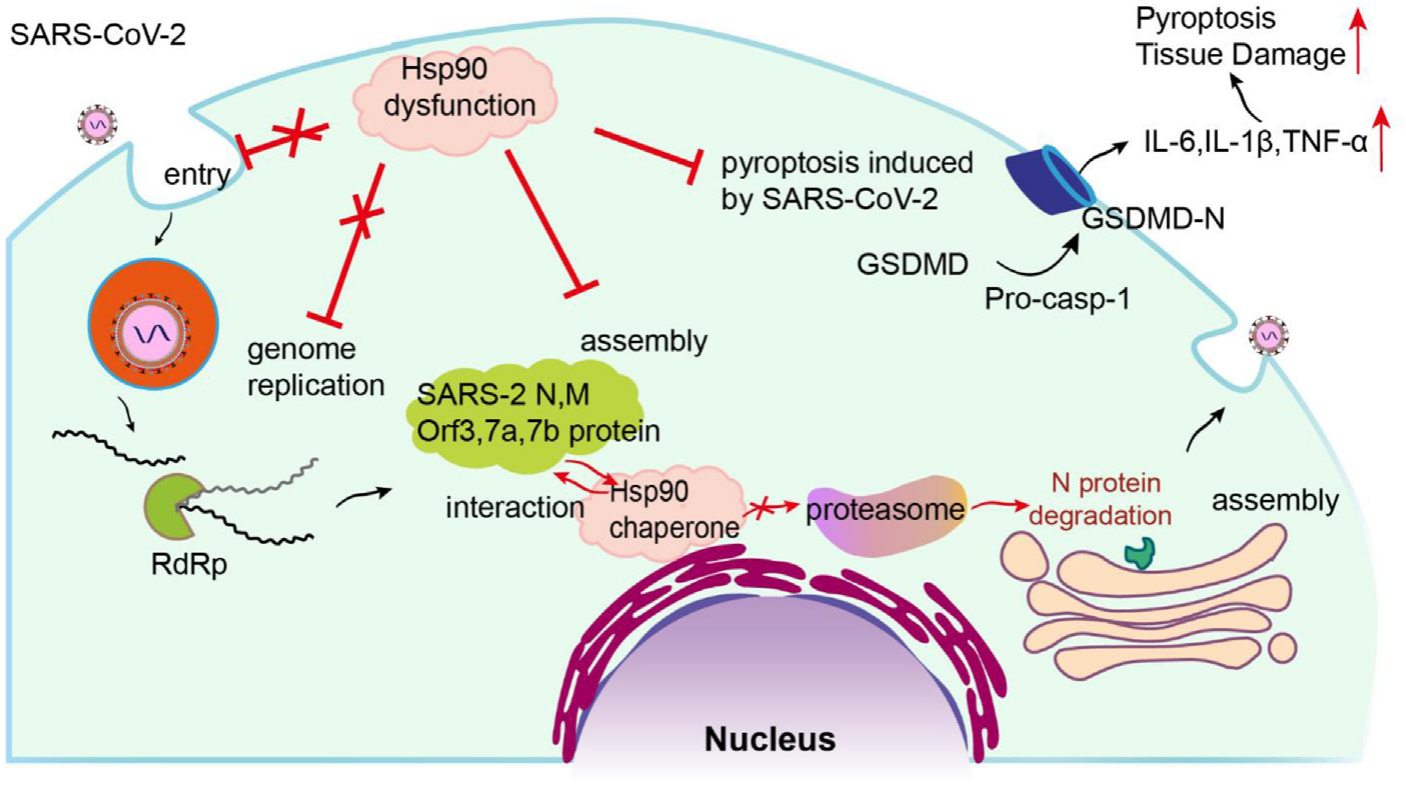
**Role of the Hsp90 network at various steps in the SARS-CoV-2 life cycle.** Hsp90 was neither shown to chaperone the SARS-CoV-2 S protein, which is required for viral entry into target cells, nor did it chaperone the nonstructural proteins needed for viral replication. Hsp90 was found to chaperone N, M, Orf3, Orf7a, and Orf7b, and regulate virion assembly by preventing proteasomal degradation of N protein. Since SARS-CoV-2 infection promotes cleavage activation of caspase-1 and GSDMD, targeting Hsp90 can inhibit this process, reducing the subsequent hyperinflammation and pyroptosis that contribute to severe COVID-19 disease. COVID-19, coronavirus disease 2019; Hsp90, heat shock protein 90; SARS-CoV-2, severe acute respiratory syndrome coronavirus 2.

In summary (Fig. 8), our study reveals the mechanism of Hsp90 in SARS-CoV-2 infection. Hsp90 interacts with multiple SARS-CoV-2 structural proteins (N, M, Orf3, Orf7a, and Orf7b). On one hand, Hsp90 dysfunction directly inhibiting virion production by affecting the proteostasis of certain SARS-CoV-2 structural proteins. On the other hand, we showed that inhibition of Hsp90 suppressed cell pyroptosis induced by SARS-CoV-2 and lung injury in infected hamsters. These new insights into the host–virus interaction of SARS-CoV-2 provide a theoretical basis for novel therapies against COVID-19.

## Experimental procedures

### Cell cultures

HEK293T, Caco-2, A549, and Huh7 cells were maintained in Dulbecco’s modified Eagle’s medium (DMEM; Hyclone) supplemented with 10% (v/v) fetal bovine serum (FBS; Biological Industries), 100 IU/ml penicillin, and 100 μg/ml streptomycin. THP-1 cells, a human monocytic cell line, were cultured in RPMI 1640 medium (Gibco) containing 10% FBS (Gibco). For differentiation into macrophages, cells were incubated with 20 nM phorbol 12-myristate 13-acetate (PMA). After 24 h, the medium was changed and cells were cultured for another 24 h without PMA. All cells were cultured at 37 °C with 5% CO_2_.

### Stable cell lines

HEK293T cells seeded in a 6-well cell culture cluster were cotransfected with psPAX2 packaging plasmid, pMD2.G envelope plasmid, and pLKO.1 shRNA-Hsp90α/β plasmid or pLenti EF1a-SARS2 N-flag, pLenti EF1a-ACE2. Culture medium containing virus was collected 48 h post-transfection (hpt), passed through a 0.45-μm filter to remove debris, and added to the target cells. Infected cells were selected with puromycin or blasticidin.

We used a CRISPR/Cas9 system (59) to generate Hsp90α-KO, Hsp90β-KO, and CHIP-KO HEK293T cells. HSP90α, HSP90β, and CHIP primer sequences (hHSP90α, 5′-CACCGCCAGACCCAAGACCAACCGA-3′, 5′-AAACTCGGTTGGTCTTGGGT CTGGC-3′; hHSP90β, 5′-CACCGCAGAGTACCTAGAAGAGAGG-3′, 5′-AAACC CTCTCTTCTAGGTACTCTGC-3′; hCHIP, 5′- GAGGTTGGCTGACAAGCTGC-3′, 5′- AGCCGGTCAGAGATGGACCT-3′) were used to amplify and clone into the plasmid guide RNA . These constructs were transfected along with Cas9-2A-GFP into HEK293 cells. Thirty-six hours after transfection, cells with green fluorescence were then sorted with a flow cytometer (BD FACS Aria II), and cell pools were identified by immunoblotting with anti-HSP90α, anti-HSP90β, or anti-CHIP antibodies.

### Reagents and antibodies

Alvespimycin (17-DMAG) HCl (cat. no. 467214-21-7), MG-132 (cat. no. 1211877-36-9), and Remdesivir (cat. no. 1809249-37-3) were purchased from Selleck Chemicals. 3-methyladenine (HY-19312) and VER-155008 (HY-10941) were purchased from MedChemExpress (shanghai, China). Bafilomycin A1 (ab120497) was purchased from Abcam. Chloroquine (CAS 50-63-5), PMA (P1585), Nigericin (481990), LPS (L2630) were purchased from Sigma. Calcein/propidium iodide (PI) Cell Viability/Cytotoxicity Assay Kit (Cat: C2015M), Hoechst 33342 (Cat: C1027), and 4′,6-diamidino-2-phenylindole (Cat: P0131) were purchased from Beyotime. CCK-8 Cell Counting Kit (A311-01) was purchased from Vazyme. CytoTox 96 Nonradioactive Cytotoxicity Assay (G1780) was purchased from Promega.

Myc-tag (71D10, #2278), myc-tag (9B11, #2276), Hsp90α (D1A7, #8165) rabbit monoclonal antibody, Hsp90β antibody (#5087), Hsp70 antibody (#4872), GSDMDantibody (#39754), and anti-β-actin (8H10D10, #3700) mouse monoclonal antibody were purchased from Cell Signaling. GAPDH (#FD0063) antibody was purchased from Fudebio-tech. Caspase-1 antibody (ab207802) was purchased from Abcam. Mouse ANTI-FLAG M2 monoclonal antibody (F3165) was purchased from Sigma. Anti-SARS-CoV-2 (2019-nCoV) N protein (Cat: 40588-R001) and anti-SARS-CoV-2 (2019-nCoV) S protein (Cat: 40591-T62) were purchased from SinoBiologica. Anti-mCherry antibody (Cat: HA500049) was purchased from HUABIO antibodies. Alexa Fluor 488- or 647-conjugated goat anti-rabbit IgG (Thermo Fisher Scientific). Dynabeads Protein G for immunoprecipitation (10004D) was purchased from Thermo Fisher Scientific.

### Plasmids

The complete coding region of each SARS-CoV-2 protein was molecularly cloned from the complementary DNA of China Institute of Veterinary Drug Control-HB-01 cell isolate (kindly provided by Dr Tan Wenjie, China CDC). NSP1-16, structural proteins (S, E, M, and N), and accessory proteins (Orf3, Orf6, Orf7a, Orf7b, Orf8, and Orf9b) were individually cloned into pRK5-myc (Promega) using EcoRI and XbaI restriction sites and confirmed by DNA sequencing. Cloning of full-length NSP3 was not successful, so it was separated into its PLpro and ADP-ribose domains. Proper expression of each protein was confirmed by Western blot. The exact positions of the genes within the viral genome and their cloning primers are listed in Table S1.

### SARS-CoV-2 GFP/ΔN virus production and infection

SARS-CoV-2 GFP/ΔN virus at passage 3 (p3) and Caco-2-N were kindly provided by Dr Qiang Ding (Tsinghua University). Caco-2-N cells were seeded into T-75 flasks, and after 16 h, the cells were infected with SARS-CoV-2 GFP/ΔN virus P3. When cytopathic effect was evident, culture medium containing the virus was collected, and cell debris was removed by centrifugation. Virus stocks were stored at −80 °C until use. SARS-CoV-2 GFP/ΔN virus at P5 used in this study was cultured in Caco-2-N cells. Virus titers were determined by endpoint dilution as 50% tissue culture ID_50_ on Caco-2-N cells. THP-1-ACE2 macrophages were infected with SARS-CoV-2 GFP/ΔN virus at an MOI = 10 for 4 h, followed by washing to remove extracellular viruses. Cells were further incubated with 10 μM nigericin for 1 h as inflammasome activation signal, followed by either cell harvest for immunoblotting or fixing for immunostaining. LPS, a well-known inflammasome activator, was used as a positive control. THP-1-ACE2 macrophages were challenged with 1 μg/ml LPS for 3 h and 10 μM nigericin for 30 min, followed by either cell harvest for immunoblotting or fixing for immunostaining.

### Immunofluorescence assay and quantitative real-time PCR

HEK293T cell lines including Hsp90α^−/−^ (90α-KO) and Hsp90β^−/−^ (90β-KO) were grown on slides, washed 2 times with PBS, fixed with 4% paraformaldehyde in PBS for 20 min, and then permeabilized with 0.5% Triton X-100 for 10 min. Primary antibody (anti-myc-tag or anti-FLAG M2, diluted 1:1000 in PBS) was added to cells and incubated for 1 h at 37 °C. Cells were washed twice with PBS and an Alexa Fluor 488- or 647-conjugated goat anti-rabbit IgG (Thermo Fisher Scientific) was added as secondary antibody, followed by 4′,6-diamidino-2-phenylindole staining. Confocal fluorescent images were obtained on a confocal laser scanning microscope (Fluoview FV1000-IX81; Olympus).

Total RNA was extracted from cells using Trizol reagent (Thermo Fisher Scientific). qRT-PCR detection of mRNA expression levels of the target gene in HEK293T cells was performed using SYBR green fluorescent dye (KAPA SYBR FAST ABI Prism qPCR kit, Kapa Biosystems Inc). The cycle conditions were 95 °C for 3 min, followed by 40 cycles of 95 °C for 10 s, 50 °C for 30 s, and a final extension of 72 °C for 20 s. The primers used are listed in Table S1.

### Measurement of SARS-CoV-2 viral titer

The full-length SARS-CoV-2 genome was inserted into an appropriately digested pET-28a vector using two unique restriction sites, NdeI and XhoI, and then linearized with XhoI. The NSP4 gene was *in vitro* transcribed using the T7 High Efficiency Transcription Kit (TransGen Biotech Co, LTD). Standard curves were generated using dilutions of a known quantity of NSP4 transcript to allow absolute quantitation of SARS-CoV-2 RNA copy numbers in each sample.

Total RNA was extracted from culture supernatants using Trizol (Thermo Fisher Scientific) following the manufacturer’s instructions. SARS-CoV-2 RNA titer was determined by one-step qRT-PCR (TOYOBO Co, LTD) targeting the NSP4 gene with the primers: 5′-TGAAAGTTTACGCCCTGACAC-3′ and 5′-ACCACTCTAACAGAACCTTCAA-3′, and the probe FAM-CACGTTATGTGCTCATGGATGGCTCTA-MGB. Samples with a cycle threshold value <35 were considered positive based on RNA standard validation data.

### CoIP and Western blot

HEK293T cells seeded in a 6-well cell culture cluster were cotransfected with pCMV3-flag-Hsp90α/β and one of the SARS-CoV-2 plasmids (or blank vector as control) using Lipofectamine 3000 (Thermo Fisher Scientific). At 48 hpt, cells were lysed with lysis buffer (25 mM Tris–HCl, 200 mM NaCl, 10 mM NaF, 1 mM Na_3_VO_4_, 25 mM β-glycerophosphate, 1% NP40, and protease cocktail [Biotool]). Three hundred μl of supernatant was incubated with 4 μg of anti-myc-tag (Cell Signaling, 71D10, #2278), or monoclonal ANTI-FLAG M2 antibody (Sigma, F3165) overnight at 4 °C, followed by incubation with 30 μl of prewashed Dynabeads Protein G at 4 °C for 12 h with mixing. After washing, the beads were eluted with 30 μl of elution buffer. The eluents were separated by 10% or 12% SDS-PAGE and then processed for Western blot analysis.

For cellular Western blots, cells were lysed in lysis buffer, resolved by SDS-PAGE and transferred onto a polyvinylidene difluoride membrane that was subsequently blocked with Tris-buffered saline containing 3% bovine serum albumin overnight at 4 °C. Proteins were detected using the anti-myc antibody or ANTI-FLAG M2 antibody at 1:1000 dilution, followed by incubation with horseradish peroxidase-conjugated anti-rabbit IgG (1:5000 dilution; Thermo Fisher Scientific).

### RNA immunoprecipitation

Caco-2-N-flag and shRNA-Hsp90β-Caco-2-N-flag cells were infected with SARS-CoV-2 GFP/ΔN virus at MOI = 1 for 8 h and cells were lysed with lysis buffer and 300 μl of supernatant was incubated with 4 μg of monoclonal ANTI-FLAG M2 antibody overnight at 4 °C, followed by incubation with 30 μl of prewashed Dynabeads Protein G at 4 °C for 12 h with mixing. After washing, the beads were resuspended in 50 mM Tris–HCl (pH 7.5), 10 mM CaCl_2_, and 20 mg/ml proteinase K. All tubes were incubated at 58 °C for 2 h with shaking to digest the proteins. Total RNA was extracted from RIP supernatants using Trizol (Thermo Fisher Scientific) following the manufacturer’s instructions. SARS-CoV-2 RNA was determined by one-step qRT-PCR targeting the NSP4 gene.

### siRNA library of human E3 ubiquitin ligases

There are several E3 ligases that can interact with Hsp90 (Table S2). The E3 ligase siRNA library was composed of three sequence-independent siRNAs per gene target and handled according to the manufacturer’s protocol.

Hsp90β-KO-N-flag cells were seeded into a 24-well plate format for siRNA library screening and HEK293T WT-N-flag cells were seeded and used as the positive control. siRNA was used at a final concentration of 100 nM with 2 μl Lipofectamine RNAiMAX (Invitrogen) reagent. After transfection, cells were incubated with siRNA under standard mammalian cell culture conditions for 72 h then lysed and analyzed by Western blot.

### VLP production of SARS-CoV-2

SARS-CoV-2 VLPs were prepared as described (28). Briefly, HEK293T cells in 100-mm dishes were cotransfected using PEI (Life Technologies) with 10 μg each of pRK5-M, pRK5-N, pRK5-E, and/or pcDNA3.1-S, according to the manufacturer’s instructions. Cells were incubated for 5 h at 37 °C in Opti-minimal essential medium. Cells were then washed with DMEM and then DMEM containing 10% FBS was added for 48 h. The supernatants were harvested and filtered through 0.45-μm membranes, then ultracentrifuged at 210,000*g* for 2 h at 4 °C. The concentration of viral proteins was collected from each tube and analyzed by Western blot.

### Cell viability assay

HEK293T cells were seeded into 96-well tissue culture plates and incubated under standard conditions until the cells were 90% confluent. Then, the cell culture media was removed and cells were incubated with 17-DMAG at concentrations of 0, 0.5, 1, 2.5, 5 or 10 μM for 12 h, followed by detection of cell viability using a CCK-8 Cell Counting Kit. Absorbance was measured at an absorbance of 450 nm. THP-1-ACE2 cells were seeded into 24-well tissue culture plates and differentiation into macrophages, the supernatants were subjected to lactate dehydrogenase assay to assess cell death using a CytoTox 96 nonradioactive cytotoxicity assay kit (Promega). Cell pellets were used to measure cell viability by Calcein/PI Cell Viability/Cytotoxicity Assay Kit (Beyotime). Only viable cells were labeled with Calcein (green fluorescence), dead cells were labeled with PI (red fluorescence).

### Enzyme-linked immunosorbent assay

Cell culture supernatant was collected to measure the levels of IL-1β (EH001-48; ExCell Bio) and TNF-α (EH009-48; ExCell Bio) using commercial ELISA kits according to the manufacturer’s instructions.

### Experimental infection of hamsters and ethics statement

One-month-old female specific-pathogen-free Syrian hamsters were used in this study. The hamsters were raised in a sterilized room with the temperature set at 25 to 27 °C, with a 12 h daily light cycle, and fed sterilized food and water. Eight hamsters per group were inoculated with 10^8.3^ pfu/mL (in 100 μl) Ad5-N or Ad5-GFP *via* the intranasal route. Body weight was measured before infection as a baseline and monitored daily thereafter.

Animal use was approved by the ethics committee of Changchun Veterinary Research Institute (IACUC approval no. AMMS-11-2020-012). All animals were handled following guidelines for the care and use of laboratory animals set by the same committee.

### Lung histological analysis

Tissue sections were analyzed on a Leica microscope after staining with H&E and scored blindly for lung damage. Scores of 0 indicate a lesion area of 0 in the lung section. Scores of four mean the lesion area is less than 10% in the lung section. Scores of five indicate a lesion area of 10% to 50% in the lung section. Scores of 6 or 7 indicate the lesion area is greater than 50% or 80%, respectively, in the lung section. A score of 8 means the lesion area took up 100% of the lung section.

### Data analysis

Statistical analysis was performed using OriginPro software (OriginLab, Northampton, Massachusetts, USA). Data are presented as means ± SEMs. Statistical comparisons were performed by one-way ANOVA with Bonferroni’s post hoc analysis or paired and unpaired Student’s *t* tests as appropriate. *p* values ≤0.05 were considered statistically significant.

## Data availability

All relevant data are within the article.

## Supporting information

This article contains supporting information.

## Conflict of interest

The authors declare that they have no conflicts of interest with the contents of this article.
