# Supplementary material for: Heat shock protein 90 facilitates SARS-CoV-2 structural protein-mediated virion assembly and promotes virus-induced pyroptosis
Source: J Biol Chem. 2023 Apr 1;299(5):104668. doi: 10.1016/j.jbc.2023.104668 (PMC10066589; doi:10.1016/j.jbc.2023.104668)
Supplement: Supporting Table S1 [file mmc1.pdf]

Table S1. Primers used in plasmid construction.

SARS-CoV-2 Protein	Clone nt	Primers (5’-3’) for cloning into pRK5-Myc
Non-structural proteins		
Nsp1	266-805	FP: TGCACCTCGGTTCTATCGATTGAATTCGCCACCATGGAGAGCCTTGTCCCT
		RP: TGATCAGCTTCTGCTCGTCGACTCTAGACCCTCCGTTAAGCTCACGCAT
Nsp2	806-2719	FP: TGCACCTCGGTTCTATCGATTGAATTCGCCACCATGGCATACACTCGCTAT
		RP: TGATCAGCTTCTGCTCGTCGACTCTAGAACCGCCTTTGAGTGTGAAGGT
ADRP (Nsp3)	3353-3763	FP: TGCACCTCGGTTCTATCGATTGAATTCGCCACCATGGGTTATTTAAAACT
		RP: TGATCAGCTTCTGCTCGTCGACTCTAGATACACAAACTCTTAAAGAATGT
PLpro (Nsp3)	4955-6946	FP: TGCACCTCGGTTCTATCGATTGAATTCGCCACCATGGAAGTGAGGACTAT
		RP: TGATCAGCTTCTGCTCGTCGACTCTAGATATCAGTTTAGAAAAATTAGGT
Nsp4	8555-10054	FP: TGCACCTCGGTTCTATCGATTGAATTCGCCACCATGAAAATTGTTAATAATT
		RP: TGATCAGCTTCTGCTCGTCGACTCTAGACTGCAAAACAGCTGAGGT
Nsp5	10055-10972	FP: TGCACCTCGGTTCTATCGATTGAATTCGCCACCATGAGTGGTTTTAGAAAAAT
		RP: TGATCAGCTTCTGCTCGTCGACTCTAGATTGGAAAGTAACACCTGAGCATT
Nsp6	10973-11842	FP: TGCACCTCGGTTCTATCGATTGAATTCGCCACCATGAGTGCAGTGAAAAGAACAAT
		RP: TGATCAGCTTCTGCTCGTCGACTCTAGACTGTACAGTGGCTACTTTGAT
Nsp7	11843-12088	FP: TGCACCTCGGTTCTATCGATTGAATTCGCCACCATGTCTAAAATGTCAGAT
		RP: TGATCAGCTTCTGCTCGTCGACTCTAGATTGTAAGGTTGCCCTGTTGT
Nsp8	12089-12685	FP: TGCACCTCGGTTCTATCGATTGAATTCGCCACCATGGCTATAGCCTCAGAGT
		RP: TGATCAGCTTCTGCTCGTCGACTCTAGACTGTAATTTGACAGCAGAAT
Nsp9	12686-13024	FP: TGCACCTCGGTTCTATCGATTGAATTCGCCACCATGAATAATGAGCTTAGT
		RP: TGATCAGCTTCTGCTCGTCGACTCTAGATTGTAGACGTACTGTGGCAGCT
Nsp10	13025-13441	FP: TGCACCTCGGTTCTATCGATTGAATTCGCCACCATGGCTGGTAATGCAACAGAAGT
		RP: TGATCAGCTTCTGCTCGTCGACTCTAGACTGAAGCATGGGTTCGCGGAGT
Nsp12	13442-16236	FP: TGCACCTCGGTTCTATCGATTGAATTCGCCACCATGCGGGTTTGCGGTGT
		RP: TGATCAGCTTCTGCTCGTCGACTCTAGACTGTAAGACTGTATGCGGTGT
Nsp13	16237-18039	FP: TGCACCTCGGTTCTATCGATTGAATTCGCCACCATGGCTGTTGGGGCTTGT
		RP: TGATCAGCTTCTGCTCGTCGACTCTAGATTGTAAAGTTGCCACATTCCT
Nsp14	18040-19620	FP: TGCACCTCGGTTCTATCGATTGAATTCGCCACCATGGCTGAAAATGTAACAGGACT
		RP: TGATCAGCTTCTGCTCGTCGACTCTAGACTGAAGTCTTGTAAAAGTGTT
Nsp15	19621-20658	FP: TGCACCTCGGTTCTATCGATTGAATTCGCCACCATGAGTTTAGAAAATGT
		RP: TGATCAGCTTCTGCTCGTCGACTCTAGATTGTAATTTTGGGTAAAATGT
Nsp16	20659-21555	FP: TGCACCTCGGTTCTATCGATTGAATTCGCCACCATGTCTAGTCAAGCGT
		RP: TGATCAGCTTCTGCTCGTCGACTCTAGATTAGTTGTTAACAAGAACAT
Structural proteins		
S	21536-25384	FP: cggGGTACCATGTTTGTTTTTCTTGTTTTATTGCCACT
		RP: ccgCTCGAGTTAATGGTGATGGTGATGATGTGTGTAATGTAATTTGACT
E	26245-26472	FP: TGCACCTCGGTTCTATCGATTGAATTCGCCACCATGTACTCATTCGTT
		RP: TGATCAGCTTCTGCTCGTCGACTCTAGAGACCAGAAGATCAGGAACTCT
M	26523-27191	FP: TGCACCTCGGTTCTATCGATTGAATTCGCCACCATGGCAGATTCCAACGGT

		RP: TGATCAGCTTCTGCTCGTCGACTCTAGACTGTACAAGCAAAGCAATAT
N	28274-29533	FP: TGCACCTCGGTTCTATCGATTGAATTCGCCACCATGTCTGATAATGGACCCCAA
		RP: TGATCAGCTTCTGCTCGTCGACTCTAGAGGCCTGAGTTGAGTCAGCACT
Accessory proteins		
Orf3	25393- 26220	FP: TGCACCTCGGTTCTATCGATTGAATTCGCCACCATGGATTTGTTTATGAGAAT
		RP: TGATCAGCTTCTGCTCGTCGACTCTAGACAAAGGCACGCTAGTAGTCG
Orf6	27202-27387	FP: TGCACCTCGGTTCTATCGATTGAATTCGCCACCATGTTTCATCTCGTT
		RP: TGATCAGCTTCTGCTCGTCGACTCTAGAATCAATCTCCATT
Orf7a	27394-27759	FP: TGCACCTCGGTTCTATCGATTGAATTCGCCACCATGAAAATTATTCTTTTCTT
		RP: TGATCAGCTTCTGCTCGTCGACTCTAGATTCTGTCTTTCTTTTGAGTGT
Orf7b	27756-27887	FP: TGCACCTCGGTTCTATCGATTGAATTCGCCACCATGATTGAACTTTCATTAATT
		RP: TGATCAGCTTCTGCTCGTCGACTCTAGAGGCGTGACAAGTTTCATTAT
Orf8	27894-28259	FP: TGCACCTCGGTTCTATCGATTGAATTCGCCACCATGAAATTTCTTGTTTTCTT
		RP: TGATCAGCTTCTGCTCGTCGACTCTAGAGATGAAATCTAAAACAACAC
Orf9b	28734-28955	FP: TGCACCTCGGTTCTATCGATTGAATTCGCCACCATGGACCCCAAAATCAGCG
		RP: TGATCAGCTTCTGCTCGTCGACTCTAGATTTACCGTCACCACCACGAAT

FR: Forward Primer; RP: Reverse Primer; nt: nucleotides;

Other primer of N, M proteins.

SARS-CoV-2 Protein	Clone nt	Primers (5'-3') for cloning into pRK5-Myc
Deletion mutants		
N1-174	1-174	FP: TGCACCTCGGTTCTATCGATTGAATTCGCCACCATGTCTGATAATGGACCCCAA
		RP: TGATCAGCTTCTGCTCGTCGACTCTCCCTTCTGCGTAGAAGCCTTT
N175-246	175-246	FP: TGCACCTCGGTTCTATCGATTGAATTCGCCACCATGAGCAGAGGCGGCAGTCAAGCC
		RP: TGATCAGCTTCTGCTCGTCGACTCTAGAAGTGACAGTTTGGCCTTGTTG
N247-419	247-419	FP: TGCACCTCGGTTCTATCGATTGAATTCGCCACCATGAAGAAATCTGCTGCTGAGGC
		RP: TGATCAGCTTCTGCTCGTCGACTCTAGAGGCCTGAGTTGAGTCAGCACT
M1-100	1-100	FP: TGCACCTCGGTTCTATCGATTGAATTCGCCACCATGGCAGATTCCAACGGT
		RP: TGATCAGCTTCTGCTCGTCGACTCTTCTGAAAGAAGCAATGAAGTAGC
M101-222	101-222	FP: TGCACCTCGGTTCTATCGATTGAATTCGCCACCATGTTTGCGCGTACGCGTTCCATG
		RP: TGATCAGCTTCTGCTCGTCGACTCTAGACTGTACAAGCAAAGCAATAT
Fluorescent tags		
N-GFP		FP1: TGCACCTCGGTTCTATCGATTGAATTCGCCACCATGTCTGATAATGGACCCCAA
		RP2: gctcacTGACCCGGCCTGAGTTGAGTCAGCACTGC
		FP3: CAGGCCGGGTCAgtgagcaagggcgaggagctgtt
		RP4: GATCAGCTTCTGCTCGTCGACTCTAGATTActtgtacagctcgtccatg
M-mcherry		FP1: TGCACCTCGGTTCTATCGATTGAATTCGCCACCATGGCAGATTCCAACGGT
		RP2: gctcacTGACCCCTGTACAAGCAAAGCAATATTGT
		FP3: GTACAGGGGTCAgtgagcaagggcgaggaggataa
		RP4: TCAGCTTCTGCTCGTCGACTCTAGATTActtgtacagctcgtccatgc
No tags		

N	FP: TGCACCTCGGTTCTATCGATTGAATTCGCCACCATGTCTGATAATGGACCCCAA
	RP: GATCAGCTTCTGCTCGTCGACTCTTTAGGCCTGAGTTGAGTCAGCAC
M	FP: TGCACCTCGGTTCTATCGATTGAATTCGCCACCATGGCAGATTCCAACGGT
	RP: TGATCAGCTTCTGCTCGTCGACTCTTTACTGTACAAGCAAAGCAATAT
E	FP: TGCACCTCGGTTCTATCGATTGAATTCGCCACCATGTACTCATTCGTT
	RP: TGATCAGCTTCTGCTCGTCGACTCTTTAGACCAGAAGATCAGGAACTCT

FR: Forward Primer; RP: Reverse Primer; nt: nucleotides;

Primer for lentivirus packaging.

Protein	Clone nt	Primers (5'-3') for cloning into pLenti-EF1α-BSD
ACE2-BSD-F		FP: TTTCAGGTGTCGTGAcGTACGggatccGCCACCatgtcaagctcttcctggctcctt
		RP: CTCTGCCCTCTCCACTGCCtgtacaggatccaaaggaggtctgaacatcatcagtgt
M-Flag-BSD-R		FP1: TTTCAGGTGTCGTGAcGTACGggatccATGGCAGATTCCAACGGTACTAT
		RP1: ATCGTCGTCATCCTTGTAATCTCTAGACTGTACAAGCAAAGCAATATTGT
		RP2: CCTCTCCACTGCCtgtacaggatccCTTATCGTCGTCATCCTTGTAATCT

Other primer of N, M proteins.

SARS-CoV-2 Protein	Clone nt	Primers (5'-3') for cloning into PB-CMV-MCS-EF1α-GreenPuro PiggyBac
No tags		
N		FP: ATAGAAGATTCTAGAGCTAGCGAATTCGCCACCATGTCTGATAATGGACCCCAA
		RP: TGAAGGAGAGATGCGAGCCCCTCGAAGCTTTTAGGCCTGAGTTGAGTCAGCACT
MYC tags		
N		FP1: ATAGAAGATTCTAGAGCTAGCGAATTCGCCACCATGTCTGATAATGGACCCCAA
		RP2: TGAAGGAGAGATGCGAGCCCCTCGAAGCTTTTACAGCAGGTCCTCCTCGCTGAT
M		FP1: CATAGAAGATTCTAGAGCTAGCGAATTCGCCACCATGGCAGATTCCAACGGTACT
		RP2: TGAAGGAGAGATGCGAGCCCCTCGAAGCTTTTACAGCAGGTCCTCCTCGCTGAT

Oligonucleotides used for RT-qPCR

Gene	
Hamster_Gapdh	FP: GCATGGCCTTCCGTGTCC
	RP: TGTCATCATACTTGGCAGGTTTCT
Hamster_TNFa	FP1: TGAGCCATCGTGCCAATG
	RP2: AGCCCGTCTGCTGGTATCAC
Hamster_IL6	FP1: GGACAATGACTATGTGTTGTTAGAA
	RP2: AGGCAAATTTCCCAATTGTATCCAG
Hamster_IL1b	FP1: TGGACCTTCCAGGATGAGGACA
	RP2: GTTCATCTCGGAGCCTGTAGTG
Human NLRP3	FP1: ATTCGGAGATTGTGGTTGGG
	RP2: AGGGCGTTGTCACTCAGGTC

Human caspase-1	FP1: CTCAGGCTCAGAAGGGAATGTC
	RP2: TGTGCGGCTTGACTTGTCC
Human TNFa	FP1: CCAGACCAAGGTCAACCTCC
	RP2: CAGACTCGGCAAAGTCGAGA
Human IL-6	FP1: AGGAGACTTGCCTGGTGAAA
	RP2: CAGGGGTGGTTATTGCATCT
Human IL-8	FP1: TTGGCAGCCTTCCTGATTTC
	RP2: CTTTAGCACTCCTTGGCAAAAC
Human IL-10	FP1: GATTTTAATAAGCTCCAAGACCAAGGT
	RP2: CTTCTATGCAGTTGATGAAGATGTCAA
Human Gapdh	FP1: GGAGTCAACGGATTTGGT
	RP2: TGATGGGATTTCCATTG
SARS-CoV-2-M	FP1: GCCACTCCATGGCACTATT
	RP2: CCTAGATGGTGTCCAGCAATAC
SARS-CoV-2-N	FP1: GTGATGCTGCTCTTGCTTTG
	RP2: GTGACAGTTTGGCCTTGTTG
FBXO10	FP1: CTGTGGCGCATGATCTTAGC
	RP2: TGGATGCAAGGTAATGCTGCT
RCBTB2	FP1: CAAGCCAGTACAGGCTACTCT
	RP2: CCGAGGTTCAATGGTGCTCTG

FR: Forward Primer; RP: Reverse Primer; nt: nucleotides;
