# Supplementary material for: Heat shock protein 90 facilitates SARS-CoV-2 structural protein-mediated virion assembly and promotes virus-induced pyroptosis
Source: J Biol Chem. 2023 Apr 1;299(5):104668. doi: 10.1016/j.jbc.2023.104668 (PMC10066589; doi:10.1016/j.jbc.2023.104668)
Supplement: Supporting Figures S1–S3 [file mmc3.pdf]

Supplementary Figure Legends

Supplementary Figure S1. Identification of the E3 ligase responsible for N protein degradation upon Hsp90β knockout by western blot. (A-H) 90β-KO-N-flag stable cells were seeded in 24-well plates for siRNA library screening. At 72 h post-transfection, cells were lysed and analyzed by western blot.

Supplementary Figure S2. Identification of the E3 ligase responsible for N protein degradation upon Hsp90β knockout by immunofluorescence. (A-B) HEK293T cells were seeded in 24-well plates, at 72 h post-transfection, cells were transfected with N-GFP plasmid for 48 h and observed by fluorescence microscopy. (C-D) 90β-KO-N-flag stable cells were seeded in 24-well plates. At 84 h post-transfection, cells were immunostained for N (green) and nuclei were stained with DAPI (blue). The cells were observed by fluorescence microscopy.

Supplementary Figure S3. Infection of Syrian hamsters by SARS-CoV-2 GFP/ΔN virus. (A) A549-ACE2 and Huh7-ACE2 cells were infected with $10^{8.5}$ PFU of Ad5-N in 100 μL for 48 h, then infected with SARS-CoV-2 GFP/ΔN virus. Lung tissue from Ad5-N/GFP-transduced hamsters was disrupted by grinding, and lysates were collected for western blot. (B) Ad5-N or Ad5-GFP-transduced hamsters were intranasally inoculated with $10^{6.5}$ PFU of SARS-CoV-2 GFP/ΔN virus in 100 μL of PBS. Body weight was monitored daily (n = 6 per group). (C) Left: SARS-CoV-2 Nsp4 mRNA copies per g of lung; right: SARS-CoV-2 M mRNA copies per g of lung were quantified by qRT-PCR. Results present mean and SD of three independent experiments.

S1 Fig A-D

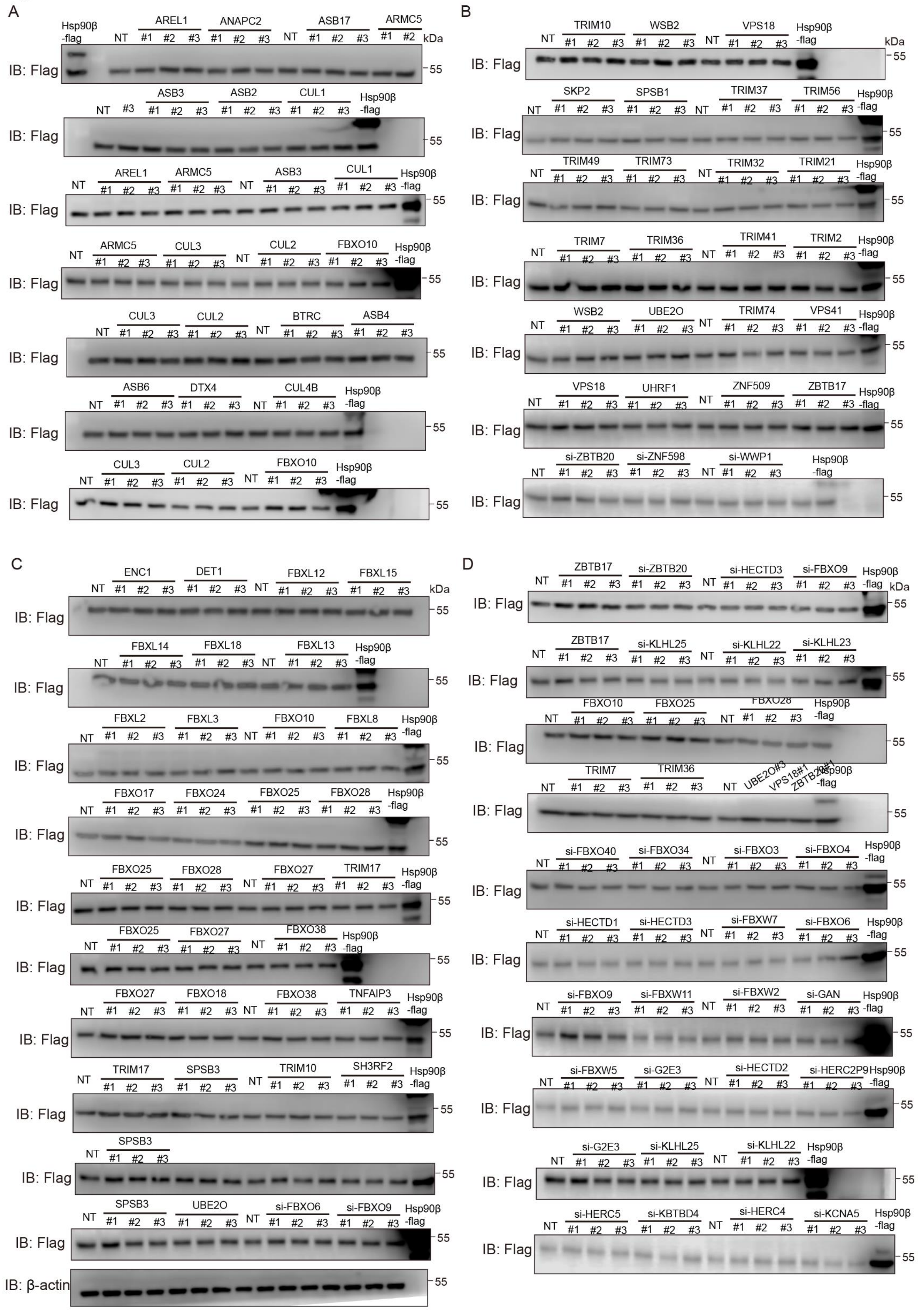

S1 Fig E-H

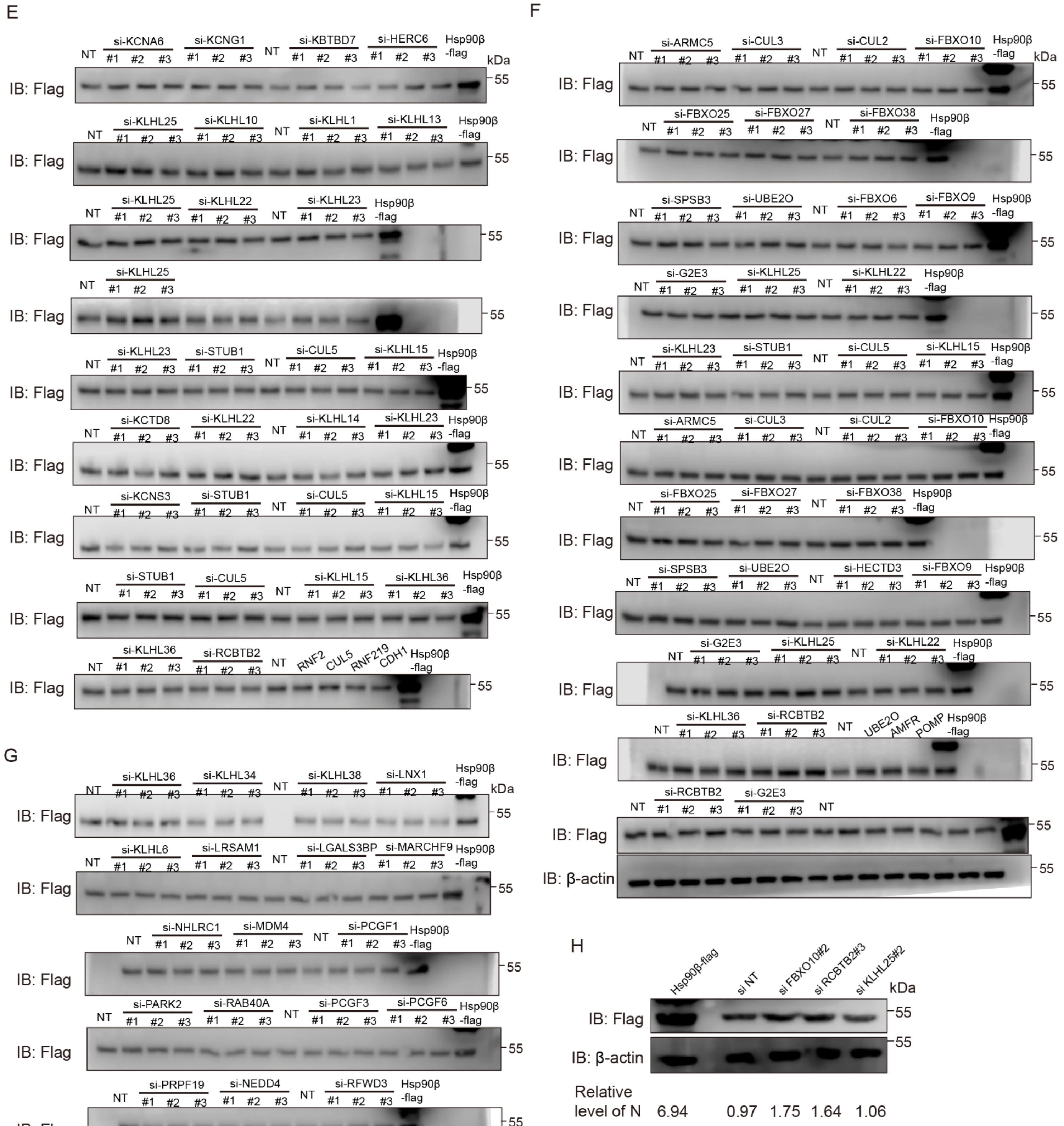
E
NT si-KCNA6 #1 #2 #3 si-KCNG1 #1 #2 #3 NT si-KBTBD7 #1 #2 #3 si-HERC6 #1 #2 #3 Hsp90β -flag
kDa
IB: Flag 55
NT si-KLHL25 #1 #2 #3 si-KLHL10 #1 #2 #3 NT si-KLHL1 #1 #2 #3 si-KLHL13 #1 #2 #3 Hsp90β -flag
IB: Flag 55
NT si-KLHL25 #1 #2 #3 si-KLHL22 #1 #2 #3 NT si-KLHL23 #1 #2 #3 Hsp90β -flag
IB: Flag 55
NT si-KLHL25 #1 #2 #3
IB: Flag 55
NT si-KLHL23 #1 #2 #3 si-STUB1 #1 #2 #3 NT si-CUL5 #1 #2 #3 si-KLHL15 #1 #2 #3 Hsp90β -flag
IB: Flag 55
NT si-KCTD8 #1 #2 #3 si-KLHL22 #1 #2 #3 NT si-KLHL14 #1 #2 #3 si-KLHL23 #1 #2 #3 Hsp90β -flag
IB: Flag 55
NT si-KCNS3 #1 #2 #3 si-STUB1 #1 #2 #3 NT si-CUL5 #1 #2 #3 si-KLHL15 #1 #2 #3 Hsp90β -flag
IB: Flag 55
NT si-STUB1 #1 #2 #3 si-CUL5 #1 #2 #3 NT si-KLHL15 #1 #2 #3 si-KLHL36 #1 #2 #3 Hsp90β -flag
IB: Flag 55
NT si-KLHL36 #1 #2 #3 si-RCBTB2 #1 #2 #3 NT RNF2 CUL5 RNF219 CDH1 Hsp90β -flag
IB: Flag 55
F
NT si-ARMC5 #1 #2 #3 si-CUL3 #1 #2 #3 NT si-CUL2 #1 #2 #3 si-FBXO10 #1 #2 #3 Hsp90β -flag
kDa
IB: Flag 55
NT si-FBXO25 #1 #2 #3 si-FBXO27 #1 #2 #3 NT si-FBXO38 #1 #2 #3 Hsp90β -flag
IB: Flag 55
NT si-SPSB3 #1 #2 #3 si-UBE2O #1 #2 #3 NT si-FBXO6 #1 #2 #3 si-FBXO9 #1 #2 #3 Hsp90β -flag
IB: Flag 55
NT si-G2E3 #1 #2 #3 si-KLHL25 #1 #2 #3 NT si-KLHL22 #1 #2 #3 Hsp90β -flag
IB: Flag 55
NT si-KLHL23 #1 #2 #3 si-STUB1 #1 #2 #3 NT si-CUL5 #1 #2 #3 si-KLHL15 #1 #2 #3 Hsp90β -flag
IB: Flag 55
NT si-ARMC5 #1 #2 #3 si-CUL3 #1 #2 #3 NT si-CUL2 #1 #2 #3 si-FBXO10 #1 #2 #3 Hsp90β -flag
IB: Flag 55
NT si-FBXO25 #1 #2 #3 si-FBXO27 #1 #2 #3 NT si-FBXO38 #1 #2 #3 Hsp90β -flag
IB: Flag 55
NT si-SPSB3 #1 #2 #3 si-UBE2O #1 #2 #3 NT si-HECTD3 #1 #2 #3 si-FBXO9 #1 #2 #3 Hsp90β -flag
IB: Flag 55
NT si-G2E3 #1 #2 #3 si-KLHL25 #1 #2 #3 NT si-KLHL22 #1 #2 #3 Hsp90β -flag
IB: Flag 55
NT si-KLHL36 #1 #2 #3 si-RCBTB2 #1 #2 #3 NT UBE2O AMFR POMP Hsp90β -flag
IB: Flag 55
NT si-RCBTB2 #1 #2 #3 si-G2E3 #1 #2 #3 NT
IB: Flag 55
IB: β-actin 55
G
NT si-KLHL36 #1 #2 #3 si-KLHL34 #1 #2 #3 NT si-KLHL38 #1 #2 #3 si-LNX1 #1 #2 #3 Hsp90β -flag
kDa
IB: Flag 55
NT si-KLHL6 #1 #2 #3 si-LRSAM1 #1 #2 #3 NT si-LGALS3BP #1 #2 #3 si-MARCHF9 #1 #2 #3 Hsp90β -flag
IB: Flag 55
NT si-NHLRC1 #1 #2 #3 si-MDM4 #1 #2 #3 NT si-PCGF1 #1 #2 #3 Hsp90β -flag
IB: Flag 55
NT si-PARK2 #1 #2 #3 si-RAB40A #1 #2 #3 NT si-PCGF3 #1 #2 #3 si-PCGF6 #1 #2 #3 Hsp90β -flag
IB: Flag 55
NT si-PRPF19 #1 #2 #3 si-NEDD4 #1 #2 #3 NT si-RFWD3 #1 #2 #3 Hsp90β -flag
IB: Flag 55
NT si-RCBTB1 #1 #2 #3 si-RHOBTB1 #1 #2 #3 NT si-RNF111 #1 #2 #3 si-RNF40 #1 #2 #3 Hsp90β -flag
IB: Flag 55
NT si-RNF19B #1 #2 #3 si-RAPSN #1 #2 #3 NT si-RNF10 #1 #2 #3 Hsp90β -flag
IB: Flag 55
NT si-RCBTB2 #1 #2 #3 si-SF3B3 #1 #2 #3 NT si-SOCS6 #1 #2 #3 si-SYVN1 #1 #2 #3 Hsp90β -flag
IB: Flag 55
H
Hsp90β-flag si NT si FBXO10#2 si RCBTB2#3 si KLHL25#2
kDa
IB: Flag 55
IB: β-actin 55
Relative level of N 6.94 0.97 1.75 1.64 1.06

S2 Fig

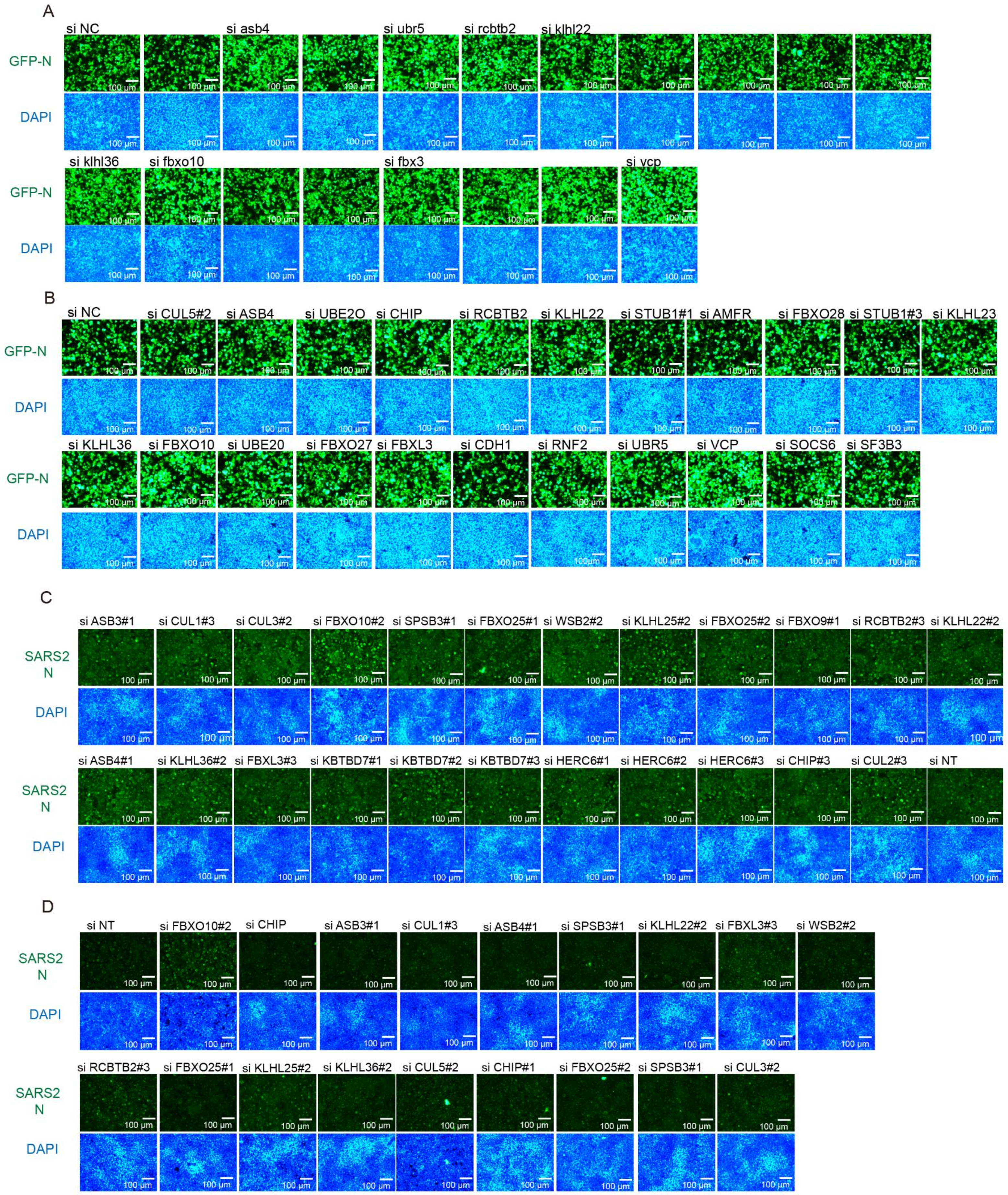
A
si NC
si asb4
si ubr5
si rcbtb2
si klhl22
GFP-N
DAPI
100 μm
si klhl36
si fbxo10
si fbx3
si vcp
B
si NC
si CUL5#2
si ASB4
si UBE2O
si CHIP
si RCBTB2
si KLHL22
si STUB1#1
si AMFR
si FBXO28
si STUB1#3
si KLHL23
si KLHL36
si FBXO10
si UBE20
si FBXO27
si FBXL3
si CDH1
si RNF2
si UBR5
si VCP
si SOCS6
si SF3B3
C
si ASB3#1
si CUL1#3
si CUL3#2
si FBXO10#2
si SPSB3#1
si FBXO25#1
si WSB2#2
si KLHL25#2
si FBXO25#2
si FBXO9#1
si RCBTB2#3
si KLHL22#2
SARS2 N
si ASB4#1
si KLHL36#2
si FBXL3#3
si KBTBD7#1
si KBTBD7#2
si KBTBD7#3
si HERC6#1
si HERC6#2
si HERC6#3
si CHIP#3
si CUL2#3
si NT
D
si NT
si FBXO10#2
si CHIP
si ASB3#1
si CUL1#3
si ASB4#1
si SPSB3#1
si KLHL22#2
si FBXL3#3
si WSB2#2
si RCBTB2#3
si FBXO25#1
si KLHL25#2
si KLHL36#2
si CUL5#2
si CHIP#1
si FBXO25#2
si SPSB3#1
si CUL3#2

S3 Fig

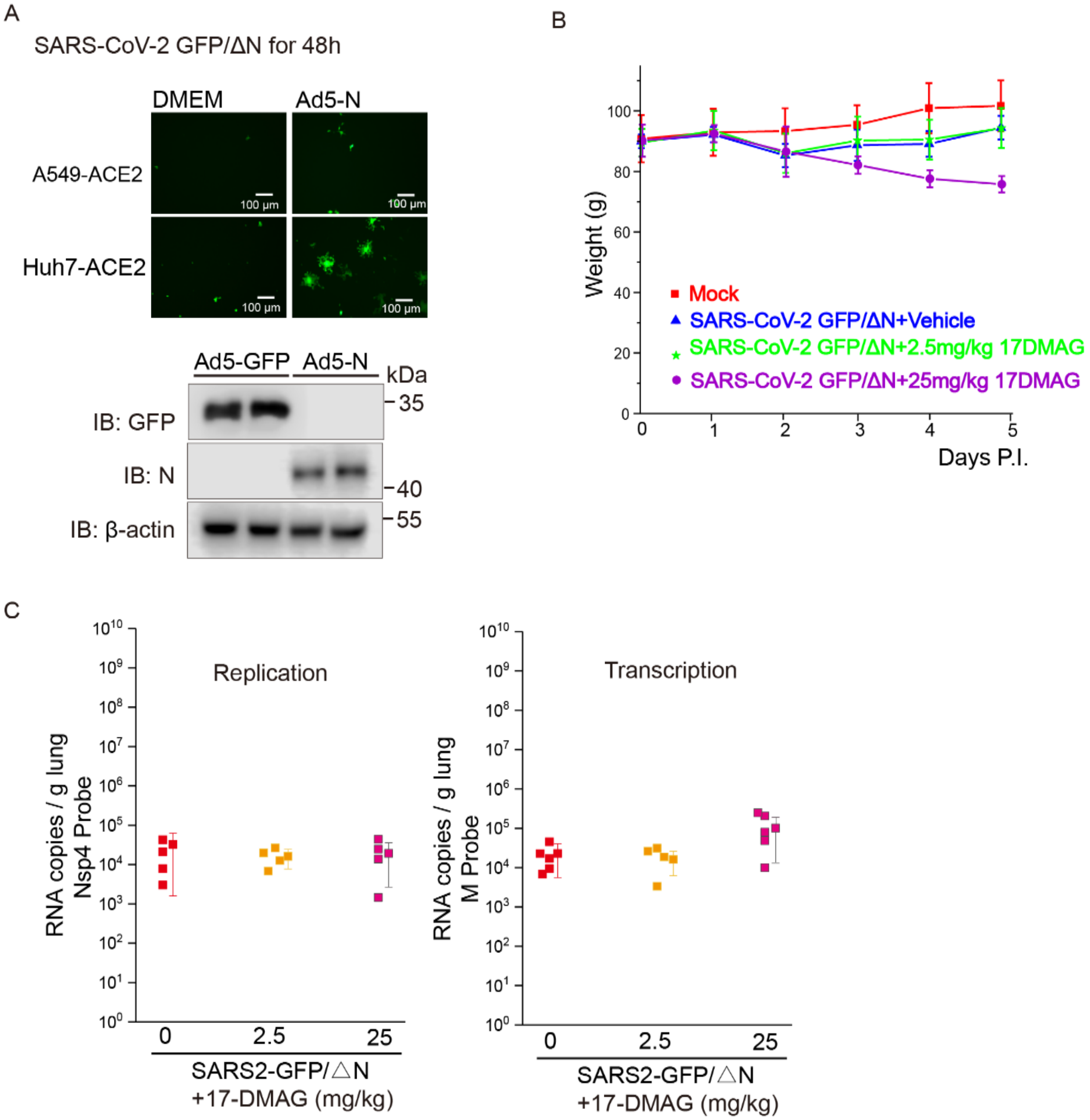
A
SARS-CoV-2 GFP/ΔN for 48h
DMEM
Ad5-N
A549-ACE2
Huh7-ACE2
100 μm
Ad5-GFP
Ad5-N
kDa
IB: GFP
IB: N
IB: β-actin
35
40
55
B
Weight (g)
Days P.I.
Mock
SARS-CoV-2 GFP/ΔN+Vehicle
SARS-CoV-2 GFP/ΔN+2.5mg/kg 17DMAG
SARS-CoV-2 GFP/ΔN+25mg/kg 17DMAG
C
Replication
RNA copies / g lung
Nsp4 Probe
SARS2-GFP/△N
+17-DMAG (mg/kg)
Transcription
RNA copies / g lung
M Probe
SARS2-GFP/△N
+17-DMAG (mg/kg)
